# One-step route to tricyclic fused 1,2,3,4-tetrahydroisoquinoline systems via the Castagnoli–Cushman protocol

**DOI:** 10.3762/bjoc.16.121

**Published:** 2020-06-24

**Authors:** Aleksandar Pashev, Nikola Burdzhiev, Elena Stanoeva

**Affiliations:** 1 Sofia University “St. Kliment Ohridski”, Faculty of Chemistry and Pharmacy, 1, James Bourchier ave., 1164 Sofia, Bulgaria; 2 Medical University Pleven, Faculty of Pharmacy, 1, St. Kliment Ohridski str., 5800 Pleven, Bulgaria

**Keywords:** benzo[*a*]quinolizidinones, Castagnoli–Cushman reaction, 3,4-dihydroisoquinolines, monocyclic anhydrides, pyrrolo[2,1-*a*]isoquinolinones

## Abstract

The Castagnoli–Cushman reaction of 3,4-dihydroisoquinolines with glutaric anhydride, its oxygen and sulfur analogues was investigated as a one-step approach to the benzo[*a*]quinolizidine system and its heterocyclic analogs. An extension towards the pyrrolo[2,1-*a*]isoquinoline system was achieved with the use of succinic anhydride. The results are evidence of an unexplored method for the access of the aforementioned tricyclic annelated systems incorporating a bridgehead nitrogen atom. The structures and relative configurations of the new compounds were established by means of 1D and 2D NMR techniques. The reactions between 1-methyldihydroisoquinoline and glutaric, diglycolic and succinic anhydrides yielded unexpected isoquinoline derivatives containing an exocyclic double bond. The compounds prepared bear the potential to become building blocks for future synthetic bioactive molecules.

## Introduction

**Figure 1:**
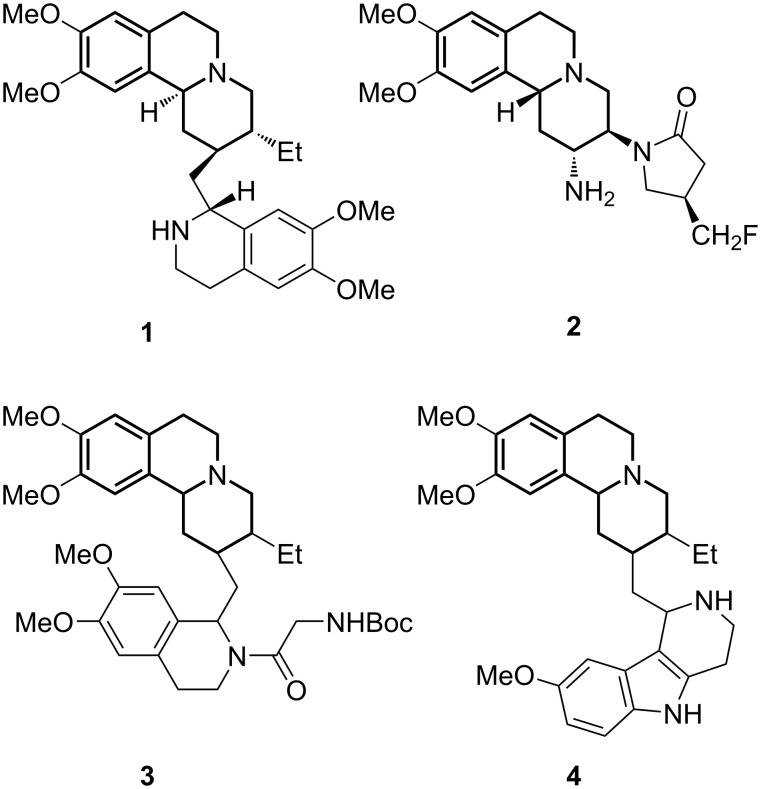
Compounds comprising a benzo[*a*]quinolizidine ring system.

The benzo[*a*]quinolizidine ring system is an important heterocyclic framework found in natural products and prospective pharmaceuticals [1]. This heterocycle is present in several alkaloids such as emetine (**1**) and related compounds, that exhibit biological activities such as glucosidase inhibition, anti-amoebic properties as well as activity against breast cancer cell lines [2]. Notable examples of synthetic compounds include the dipeptidyl peptidase IV inhibitor carmegliptin (**2**, DPP IV) with potential for the treatment of type-II diabetes [3–4]. A comparative study on novel classes of anticancer drugs identified benzo[*a*]quinolizines **3** and **4** (Figure 1) to be useful for a specific inhibition of heat shock response in cancer cells, which strongly enhances the treatment by sensitizing cancer cells to anticancer drugs [5]. The presence of such structural pattern has driven the development of various approaches for its obtaining – based either on isolation from naturally occurring sources or through multistep synthetic routes [6].

The pyrrolo[2,1-*a*]isoquinoline skeleton is incorporated in a large group of natural compounds such as crispines, trolline, and lamellarins, which are interesting due to their anticancer, antiviral, and antibacterial activities [7–8]. In the light of this broad array of biological activities, the development of novel methods for the construction of these heteropolycycles is of great significance to both organic and medicinal chemistry.

**Scheme 1:**
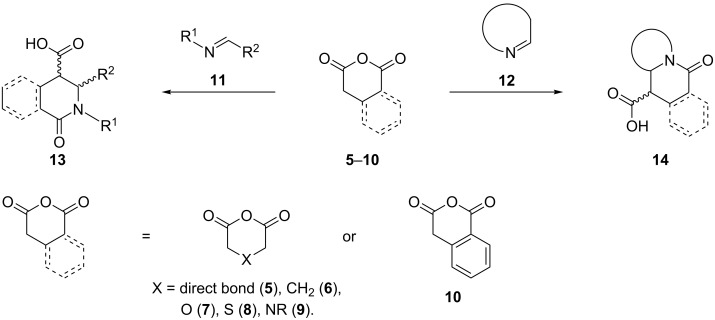
Reactions between enolizable anhydrides and imines.

The Castagnoli–Cushman reaction (CCR) between cyclic enolizable anhydrides (such as succinic (**5**) [9], glutaric (**6**) [10], and the corresponding oxygen (**7**) [11], sulfur (**8**) [11], and nitrogen analogs **9** [12], and homophthalic anhydride (**10**) [13–14]) and imines **11**, **12** offers a route to substituted lactam molecules **13** and **14** [13–19]. Among them, the reactions of homophthalic anhydride (**10**) with Schiff bases **11** or cyclic imines **12** have been examined in detail (Scheme 1) [13–14,20–21].

**Scheme 2:**
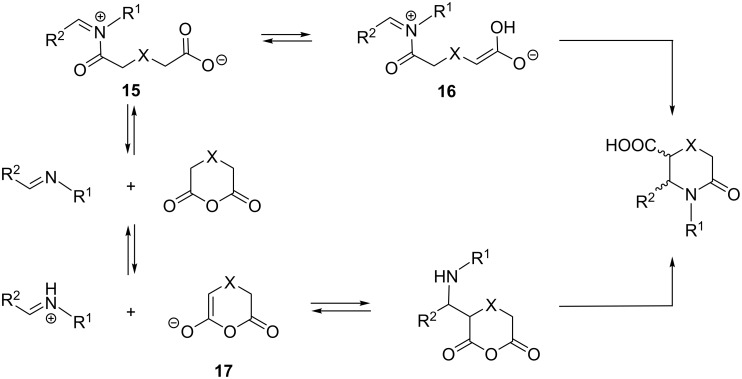
Mechanistic pathways for the reaction between cyclic anhydrides and imines.

The mechanism of the reaction is still under debate with two prevailing versions in the literature (Scheme 2) [16–17,22–24]. The first reaction pathway includes the formation of an *N*-acyliminium ion **15**, followed by a ring closure through an enolate ion **16**. The other mechanistic proposal features a stepwise Mannich-type reaction of the enolized anhydride **17** to the imine component and a subsequent *N*-acylation reaction to form the lactam target product [24]. A respective Mannich-type intermediate has been recently isolated and subsequently converted into the target lactam product [24]. It is also suggested that the imine substrate structure and the anhydride’s ability to undergo enolization have a profound influence on the reaction course [16,23].

While the reactions of homophthalic anhydride (**10**) and various cyclic imines **12** have been extensively explored [13,21,24–25], there are only few reports featuring reactions between monocyclic anhydrides **5**–**9** and cyclic imines **12** in the literature [12,25–29]. Thus, it was shown that glutaconic anhydride reacts with 1-methyl-3,4-dihydroisoquinoline and with 1-chloroisoquinoline to give 11b-methylbenzo[*a*]quinolizin-4-one [28] and benzo[*a*]quinolizin-4-one [27], respectively, in moderate yields. Attempts by Krasavin et al. showed that isoquinoline itself demonstrated modest reactivity and diastereoselectivity in reactions with thiodiacetic anhydride (**8**) [12]. It can be concluded that due to their stability the aromatic heterocycles have a low tendency to undergo reactions with cyclic anhydrides, resulting in low to moderate overall yields of the target products [12,27]. Further research by Krasavin et al. on the reactivity of sterically hindered indolenines in reactions with anhydrides **6**–**9**, did not give the expected fused δ-lactams [26].

The purpose of the present investigation was to explore the much less studied reaction between monocyclic anhydrides **5**–**8** and cyclic imines (such as 6,7-dimethoxy-3,4-dihydroisoquinoline (**18**) and its 1-alkyl derivatives **19** and **20**) as a potential one-step route towards substituted benzo[*a*]quinolizidinones and pyrrolo[2,1-*a*]isoquinolinones. This reaction also would allow the construction of bioisosteric oxygen and sulfur derivatives of the target heterocyclic framework.

## Results and Discussion

**Scheme 3:**
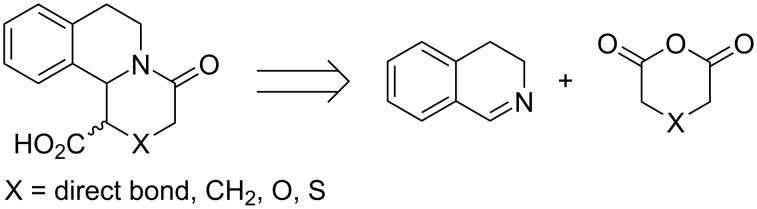
Retrosynthetic analysis of the target compounds.

Our synthetic strategy relied on the possibility to form ring C of the target ring system using the Castagnoli–Cushman reaction between 6,7-dimethoxy-3,4-dihydroisoquinoline (**18**) and its 1-substituted derivatives **19** and **20**, and the anhydrides **5**–**8**. This approach also could allow the construction of bioisosteric O- and S-analogs of the target systems as it is shown by the retrosynthetic analysis (Scheme 3). For the present studies the employed starting imines **18**–**20**, were prepared by a Bischler–Napieralski condensation, using procedures available in the literature [30–31].

### Reactions of 6,7-dimethoxy-3,4-dihydroisoquinoline (**18**) with anhydrides **5–8**

**Scheme 4:**
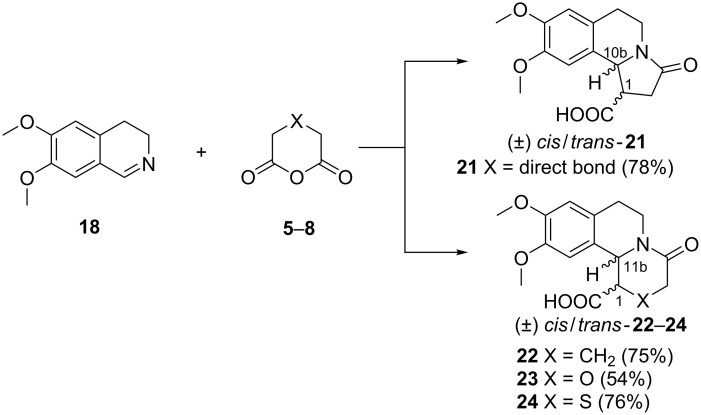
Reaction of 6,7-dimethoxy-3,4-dihydroisoquinoline (**18**) with anhydrides **5**–**8**. Reagents and conditions: xylene, inert atmosphere, 120 °C, 6 h.

The reaction between **18** and anhydrides **5**–**8** was conducted in xylene under inert atmosphere at 120 °C (Scheme 4). Previous knowledge on the reaction between monocyclic anhydrides **5**, **6** and **7** and Schiff bases indicated the necessity of elevated temperatures for conducting the reaction as the yields of the target products increased with the temperature [32–34]. The anhydride was taken in slight molar excess in analogy to our previous work [21].

The reaction mixtures were heated for 6 h, until the imine **18** reacted completely. In the course of the reaction two stereocenters were formed at C-10b for **21** and C-11b for **22**–**24**, respectively**,** and C–1. It should be noted that the compounds described here are racemic. After removal of xylene the ^1^H NMR spectrum of the residue was taken to give the diastereomeric *cis*:*trans* ratio, which in the reaction mixtures was 1:1. The obtained mixtures of diastereomers **21**–**24** were separated by column chromatography to give the individual diastereomers. The acid (−)-*trans*-**22** and its methyl ester are known compounds, which were prepared by an independent method [35].

**Figure 2:**
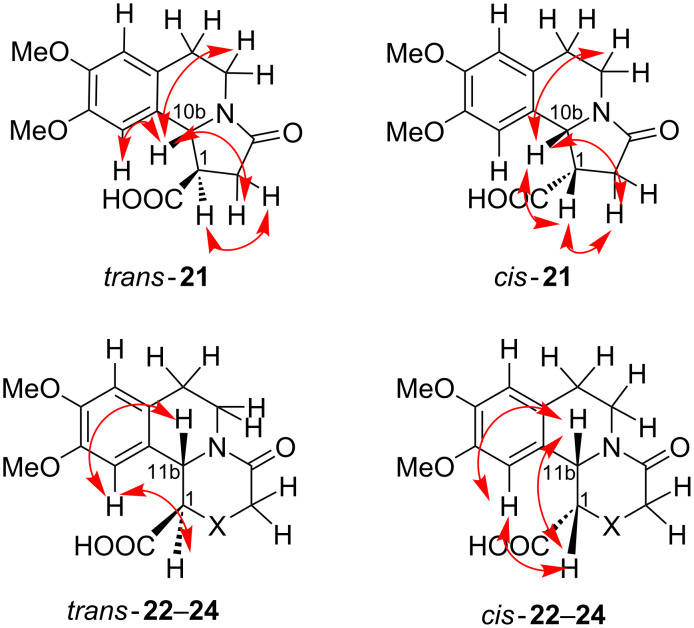
Representative NOE interactions in *cis* and *trans-***21**–**24** (only one enantiomer is shown).

NOESY experiments were used to examine the stereochemical assignments in compounds **21**–**24**. The *cis* diastereomers **22**–**24** showed distinct NOE cross-signals between protons H-1 and H-11b, and for *cis*-**21** – between H-1 and H-10b. No such NOE peaks were identified in the spectra of the *trans* isomers (Figure 2). Further, the assignment of the relative configuration of the products **22**–**24** was supported by the vicinal coupling constants ^3^*J*_1,11b_ for H-1 and H-11b. The ^1^H NMR spectra of the *trans* diastereomers were expected to display a larger coupling constant ^3^*J*_1,11b_ in comparison with the spectra of the corresponding *cis* analogs [36]. A similar difference was observed in the spectra of the diastereomeric dibenzo[*a*,*g*]quinolizidinone compounds as well [14,37–39].

As it can be seen from Table 1, the spectra of *cis*-**22**–**24** displayed ^3^*J*_1,11b_ within the narrow interval of 4.0–4.2 Hz, which could be attributed to a relatively more rigid conformation in the *cis* isomers. The spectra of the *trans*-**22**–**24** compounds exhibited ^3^*J*_1,11b_ in a broader interval of 5.7–10.3 Hz, which could be explained by a higher conformational flexibility of the *trans* isomers. Similar values of ^3^*J*_1,11b_ were observed in the spectra of the known (−)-*trans*-**22** [35] and the closely related structural analogs *trans*-1-ethylbenzo[*a*]quinolizidin-4-ones, previously described by Amat et al. [40]. The ^1^H NMR spectra of *trans*-**21** and *cis*-**21** displayed very close values for *^3^J*_1,10b_, i.e., 6.9 Hz for *trans*-**21**, and 6.4 Hz for *cis*-**21**, which hindered the assignment of the relative configuration based solely upon the *^3^J* values. However, taking into account the above-mentioned NOESY experiments, the relative configuration of compound **21** displaying *^3^J*_1,10b_ = 6.4 Hz was assigned as *cis,* and the configuration of the compound with *^3^J*_1,10b_ = 6.9 Hz as the *trans* isomer (Table 1).

**Table 1:** NMR data and stereochemical assignment of *cis* and *trans-***21**–**24**.

	H–1, δ ppm	H-11b^a^, δ ppm	^3^*J*_1,11b_^a^, Hz

*cis-***21**	3.90–4.04 m	4.86 d	6.4 Hz
*trans-***21**	3.48 t	4.97 d	6.9 Hz
*cis-***22**	4.64 m	4.84 d	4.2 Hz
*trans-***22**	3.10 ddd	4.93 d	7.1 Hz
*cis-***23**	4.96 d	5.16 d	4.2 Hz
*trans-***23**	5.03 d	4.73 d	5.7 Hz
*cis-***24**	4.54 d	5.08 d	4.0 Hz
*trans-***24**	3.55 d	5.05 d	10.3 Hz

^a^For compounds *cis* and *trans*-**21** the values for H-10b and ^3^*J*_1,10b_ are given.

As it is known, the benzo[*a*]quinolizidine system can exist as interconvertible conformers due to the presence of a stereochemically mobile nitrogen atom [41]. Numbers of studies suggested that the preferred conformation of benzo[*a*]quinolizidines depended strongly on the type of substituents, the presence of stereocenters and their configuration [36,41–46]. As for the 4-oxobenzo[*a*]quinolizidine derivatives, it should be noted that due to the planar amide fragment the interconversion of the different conformers could be largely suppressed. The X-ray analysis of the above-mentioned (−)-*trans*-**22** [35] and *trans*-1-ethylbenzo[*a*]quinolizin-4-ones [40] showed a distortion of the B and C rings, while the substituent at C-1 acquired a spatial orientation with a smaller steric repulsion with H-11. Based on the data outlaid above the assignment of the preferred conformation of the acids *trans*- and *cis*- **21**–**24** posed significant challenge.

### Reactions of 1-alkyl-3,4-dihydroisoquinolines **19** and **20** with anhydrides **5**–**8**

**Scheme 5:**
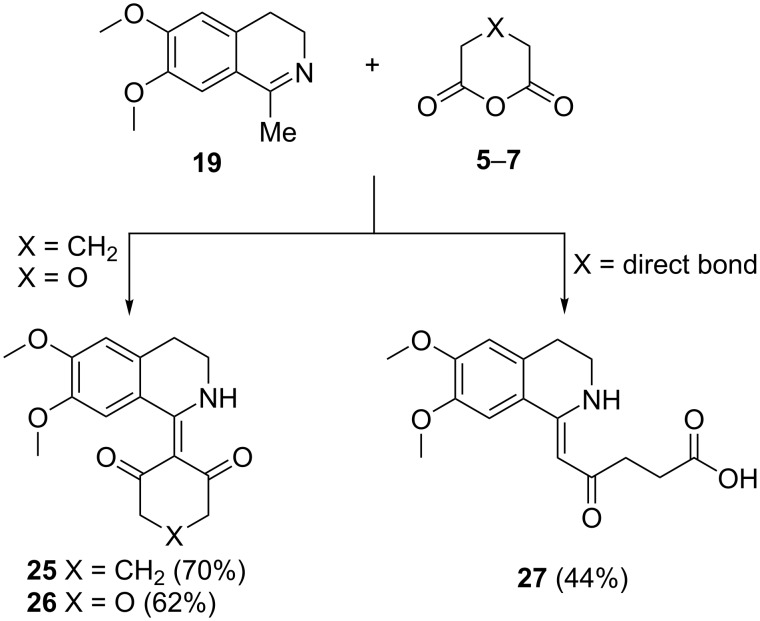
Reaction of 1-methyl-3,4-dihydroisoquinoline (**19**) with anhydrides **5**–**7**. Reagents and conditions: xylene, inert atmosphere, 120 °C, 6 h.

The reactions of 1-methyl and 1-ethyl-6,7-dimethoxy-3,4-dihydroisoquinoline **19** and **20** with anhydrides **5**–**8** were investigated under the same reaction conditions as the reactions of imine **18**. When glutaric (**6**) and diglycolic anhydride (**7**) were subjected to the reaction with **19**, the reaction yielded the unexpected products **25** and **26**, respectively, containing an exocyclic double bond to the tetrahydroisoquinoline moiety (Scheme 5). The reaction with succinic anhydride (**5**) furnished the tetrahydroisoquinoline **27** with an open-chain acid substituent to the exocyclic double bond. Unlike 1-methyldihydroisoquinoline (**19**), the 1-ethyl derivative **20** did not react at all with the anhydrides **5**–**7**.

**Figure 3:**
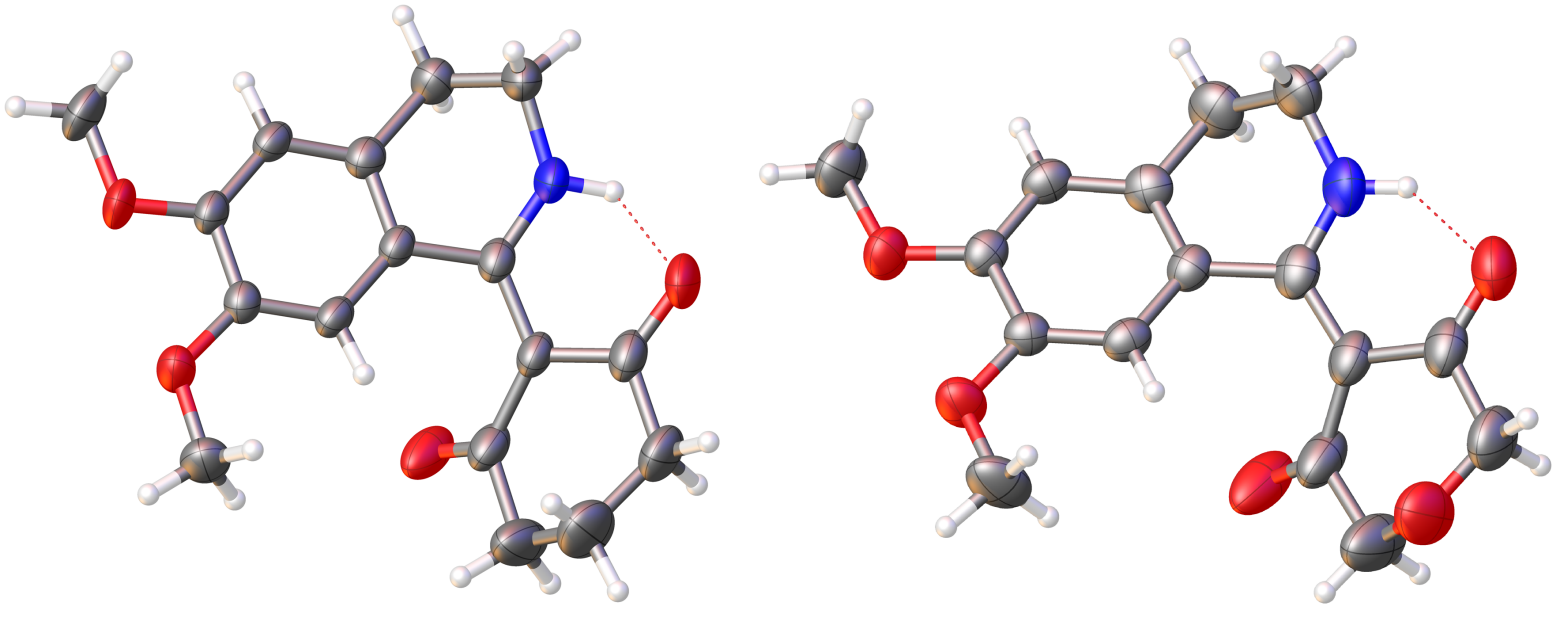
X-ray crystal structure of products **25** and **26**.

The structures of compounds **25** and **26** were confirmed by 2D NMR spectroscopy and singe-crystal X-ray analysis (Figure 3).

The piperidine moiety is planar to a significant extent due to the combined influence of the aromatic ring and the exocyclic C–C double bond. The geometry of the nitrogen atom is also planar most likely because of conjugation with the exocyclic C–C double bond and the C=O groups. The nitrogen planarity is probably also caused by a hydrogen bond between the NH group and the neighboring carbonyl oxygen atom. The structure of **27** was proven by a combination of 1D and 2D NMR spectra, and IR spectroscopy.

**Scheme 6:**
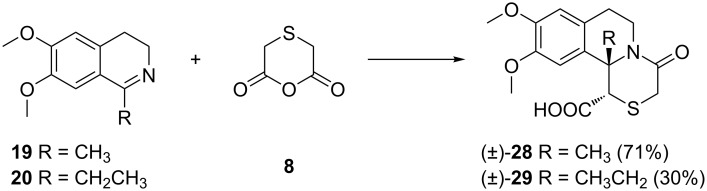
Reactions of 1-alkyl-3,4-dihydroisoquinolines **19** and **20** with anhydride **8**. Reagents and conditions: xylene, inert atmosphere, 120 °C, 6 h.

The reactivity of thiodiacetic anhydride **8** towards the imines **19** and **20** was different to that of the anhydrides **5**–**7**. The targeted 11b-methyl and 11b-ethyl-4-oxo-2-thiabenzo[*a*]quinolizidinones **28** and **29**, respectively, were formed as single diastereomers (Scheme 6). The 1-ethyldihydroisoquinoline **20** showed a pronounced lower reactivity to anhydride **8**, with a 30% yield of **29** as compared to **28** (71%), probably due to a steric hindrance.

**Figure 4:**
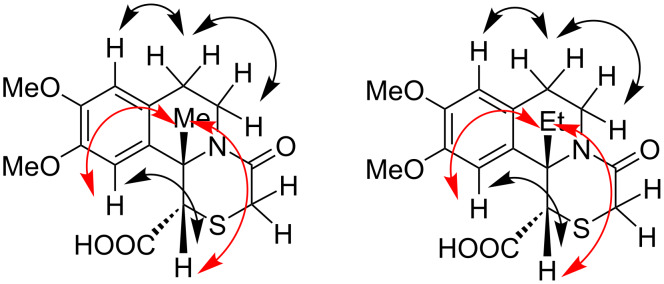
Representative NOE interactions in **28** and **29**.

The relative configuration of **28** and **29** was established using 2D ROESY NMR spectroscopy. The spectra showed cross peaks between the protons from the *cis*-oriented alkyl-C-11b and H-1, which unequivocally proved the *trans* relative configuration of the substituents at C-1 and C-11b (Figure 4).

1-Alkyl-3,4-dihydroisoquinolines have been reported to react as C-nucleophiles or 1,3-C,N-ambident nucleophiles with electrophilic species [47–50].

**Scheme 7:**
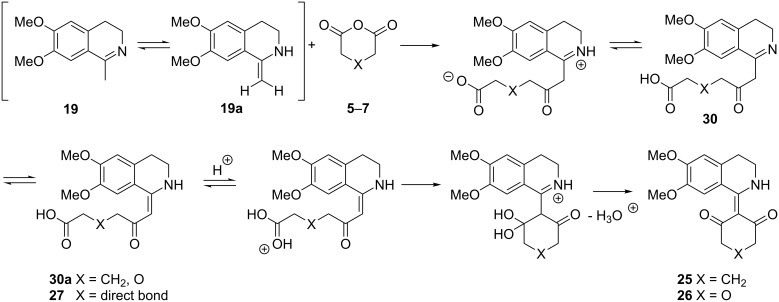
Suggested mechanism for the formation of products **25**–**27**.

Thus the formation of compounds **25**–**27** could be rationalized as including an initial nucleophilic attack of the enamine tautomer **19a** of 3,4-dihydroisoquinoline **19** to the carbonyl carbon atoms of the anhydrides **5**–**7** followed by an anhydride ring opening. In the case of anhydrides **6** and **7**, a 6-*exo-trig* ring-closure reaction of the tautomer form **30a** leads to the observed products **25** and **26,** according to the plausible mechanistic suggestion presented in Scheme 7.

In the case of compound **27** (X = direct bond), the reaction could be characterized as a 5-*exo-trig* cyclization, which is favored as long as the Bürgi–Dunitz angle is achievable [51]. It could be concluded that the reaction does not proceed further probably because the shorter side chain makes difficult the achievement of the required angle of approach of the nucleophile.

The observed different reactivity of the anhydrides **5**–**7** towards 1-methyl-3,4-dihydroisoquinoline **19** needs additional explanation. Work in this field based on the theoretical treatment of the possible stabilities of the intermediates and the hypothetic transition states of the steps is in progress.

## Conclusion

The scope of the Castagnoli–Cushman reaction was enlarged by the use of 3,4-dihydroisoquinolines and succinic, glutaric, diglycolic, and thiodiacetic anhydrides. The substituted benzo[*a*]quinolizidine products and their heterocyclic analogs as well as 3-oxopyrrolo[2,1-*a*]isoquinolin-1-carboxylic acids were obtained as a 1:1 mixture of diastereomers with varying yields when 3,4-dihydroisoquinoline was used. The reaction of 1-methyl and 1-ethyldihydroisoquinoline with thiodiacetic anhydride afforded the expected angularly substituted *trans*-2-thiabenzo[*a*]quinolizidinones. On the contrary, the glutaric, succinic, and diglycolic anhydrides reacted with 1-methyldihydroisoquinoline to give isoquinoline products with an exocyclic double bond. This research demonstrated once more the versatility of 3,4-dihydroisoquinolines as synthons for the preparation of fused polyheterocycles containing a bridgehead nitrogen atom.

## Experimental

### General

Melting points were taken on an automated melting point apparatus SRS EZ-Melt MPA120 at a ramp rate of 1 °C/min and are uncorrected. IR spectra were recorded on a Thermo Fischer Nicolet iS50 FT-IR instrument. Spectroscopic grade KBr was used in the sample preparation without further purification. ^1^H NMR spectra (500 MHz) and ^13^C NMR spectra (125 MHz) were obtained on a Bruker Avance III HD 500 MHz spectrometer. The chemical shifts are given in parts per million (δ) for the spectra in DMSO-*d*_6_ relative to the residual solvent peak [52]. Coupling constants (*J*) are reported in Hz. Assignments were made by using a combination of 1D and 2D spectra (COSY, HSQC and HMBC). Thin-layer chromatography (TLC) was performed on Merck 1.05554 silica gel 60F254 aluminum plates. Chromatographic filtration and column chromatography were carried out using Merck Kiesegel 60 (0.060–0.200 mm). High-resolution mass spectra (HRMS) were acquired on a Thermo Scientific, model Q-Exactive high resolution LC–MS/MS with a resolution of up to 150000FWHM at *m*/*z* 200.

X-ray structure determination: Colorless single crystals of compounds **25** and **26** were obtained from DMSO/H_2_O. Diffraction data were collected at 300 K the by v-scan technique, on a Mitigen Micromount and automatically centered on a Bruker SMART X2S Benchtop Crystallographic system. Intensity measurements were performed using monochromatized Mo Kα radiation (λ = 0.71073 Å) from a sealed microfocus tube and BREEZE CCD detector. The APEX 2 v.2014.11.0 software was used for all data processing [53]. The structure was solved and refined with SHELX [54] programs, ShelXT and ShelXL by using OLEX 2 software [55]. Hydrogen atoms were placed at idealized positions and refined. Crystallographic data (excluding structure factors) for the structural analysis were deposited with the Cambridge Crystallographic Data Centre, CCDC 1963825 and 1963826, respectively. Copy of this information can be obtained free of charge from The Director, CCDC, 12 Union Road, Cambridge, CB2 1EZ, UK. Fax: +44 1223 336–033, e-mail: deposit@ccdc.cam.ac.uk, or http://www.ccdc.cam.ac.uk.

### General procedure for preparation of (±)-*trans* and *cis*-**21**–**24**

The starting 6,7-dimethoxy-3,4-dihydroisoquinolinium perchlorate (1.2 mmol, 0.35 g) was dissolved in water (50 mL) and the resulting suspension was treated with solid sodium hydroxide until pH 10. The resulting mixture was extracted with dichloromethane (3 × 10 mL) and the organic phase was dried over anhydrous sodium sulfate and evaporated under reduced pressure. The obtained free base (0.21 g, 92%, 1.1 mmol) was dissolved in dry xylene (2.5 mL) and the corresponding anhydride (1.4 mmol) was added. The reaction mixture was heated at 120 °C under inert atmosphere for 6 hours. TLC was used to monitor the course of the reaction. Upon completion, the solvent was evaporated under reduced pressure and the resulting crude products were separated and purified by means of column chromatography. The ratio of the *cis*:*trans* diastereomers was obtained from the ^1^H NMR spectra of the crude reaction mixture.

### General procedure for preparation of **25**–**28**

The corresponding 1-methyl or 1-ethyl-3,4-dihydroisoquinoline (**19**, 1 mmol, 0.205 g; **20**, 1 mmol, 0.219 g) was dissolved in dry xylene (2 mL) and the corresponding anhydride (1.5 mmol) was added. The reaction mixture was heated under inert atmosphere for 6 hours. TLC was used to monitor the course of the reaction. After completion, the solvent was evaporated under reduced pressure and the resulting crude products were isolated and purified by means of column chromatography, followed by recrystallization.

## Supporting Information

File 1 Experimental procedures for compounds **21**–**29** and their spectroscopic and analytic data.
