# Supplementary material for: One-step route to tricyclic fused 1,2,3,4-tetrahydroisoquinoline systems via the Castagnoli–Cushman protocol
Source: Beilstein J Org Chem. 2020 Jun 24;16:1456–64. doi: 10.3762/bjoc.16.121 (PMC7323625; doi:10.3762/bjoc.16.121)

BEILSTEIN JOURNAL OF ORGANIC CHEMISTRY

Supporting Information

for

One-step route to tricyclic fused 1,2,3,4-tetrahydroisoquinoline systems via the Castagnoli–Cushman protocol

Aleksandar Pashev, Nikola Burdzhiev and Elena Stanoeva

Beilstein J. Org. Chem. **2020,** *16,* 1456–1464. doi:10.3762/bjoc.16.121

Experimental procedures for compounds 21–29 and their spectroscopic and analytic data

License and Terms: This is a supporting information file under the terms of the Creative Commons Attribution License (http://creativecommons.org/licenses/by/4.0). Please note that the reuse, redistribution and reproduction in particular requires that the authors and source are credited.
The license is subject to the *Beilstein Journal of Organic Chemistry* terms and conditions: (https://www.beilstein-journals.org/bjoc)

Table of contents

Preparation of *cis* and *trans*-**21** S2
Preparation of *cis* and *trans*-**22** S3
Preparation of *cis* and *trans*-**23** S4
Preparation of *cis* and *trans*-**24** S5
Preparation of **25** S6
Preparation of **26** S7
Preparation of **27** S7
Preparation of **28** S8
Preparation of **29** S8
^{1}H and ^{13}C NMR spectra of *cis*-**21** S10
^{1}H and ^{13}C NMR spectra of *trans*-**21** S11
^{1}H and ^{13}C NMR spectra of *cis*-**22** S12
^{1}H and ^{13}C NMR spectra of *trans*-**22** S13
^{1}H and ^{13}C NMR spectra of *cis*-**23** S14
^{1}H and ^{13}C NMR spectra of *trans*-**23** S15
^{1}H and ^{13}C NMR spectra of *cis*-**24** S16
^{1}H and ^{13}C NMR spectra of *trans*-**24** S17
^{1}H and ^{13}C NMR spectra of **25** S18
^{1}H and ^{13}C NMR spectra of **26** S19
^{1}H and ^{13}C NMR spectra of **27** S20
^{1}H and ^{13}C NMR spectra of *trans*-**28** S21
^{1}H and ^{13}C NMR spectra of *trans*-**29** S22

(±)-*trans*- and *cis*-8,9-Dimethoxy-3-oxo-1,2,3,5,6,10b-hexahydropyrrolo[2,1-*a*]isoquinoline-1-carboxylic acid (21): Obtained from 3,4-dihydroisoquinoline (**18**) and 0.14 g succinic anhydride (**5**). Purification by means of column chromatography (ethyl acetate/light petroleum/formic acid 1:1:0.1) followed by recrystallization (ethyl acetate) yielded the two diastereomers as white solids. Total yield is 0.250 g, 78%.

cis-**21**: Isolated 0.152 g (48%). Mp 149.4-151.1°C. IR (KBr): 3300-2400 (OH), 1710 (CO), 1675 (CON) cm^{-1}. ^{1}H-NMR δ (DMSO-d_6): 2.57 (1H, m, H-6); 2.64 (2H, m, H-5); 2.72 (1H, m, H-6); 3.01 (2H, m, H-2); 3.69 (3H, s, CH_3O); 3.72 (3H, s, CH_3O); 4.02 (1H, m, H-1); 4.87 (1H, d, H-10_b, *J*=6.4 Hz); 6.73 (1H, s, H-7); 6.88 (1H, s, H-10), 13.02 (1H, br. s., COOH). ^{13}C-NMR δ (DMSO-d_6): 27.4 (1C, C-6); 35.0 (1C, C-2); 35.0 (1C, C-5); 36.70 (1C, C-1); 55.4 (1C, CH_3O); 55.5 (1C, CH_3O); 58.22 (1C, C-10_b); 108.5 (1C, C-10); 112.2 (1C, C-7); 126.0 (1C, C-6_a); 128.7 (1C, C-10_a); 147.59 (1C, C-8); 147.71 (1C, C-9); 169.87 (1C, C-3); 174.8 (1C, COOH). HRMS: Calc. for $C_{15}H_{18}NO_5$ $[M+H]^+$ 292.1184; found 292.1184.

trans-**21**: Isolated 0.096 g (30%) Mp 153.5-155.4°C IR (KBr): 3300-2500 (OH), 1709 (CO), 1674 (CON) cm^{-1}. ^{1}H-NMR δ (DMSO-d_6): 2.30 (1H, d, H-2, *J*=16.1 Hz); 2.58 (2H, m, H-6); 2.67 (1H, m, H-2); 2.83 (1H, m, H-5), 3.48 (1H, t, H-1, *J*=7.4 Hz); 3.70 (6H, CH_3O); 4.11 (1H, m, H-5); 4.97 (1H, d, H-10_b, *J*=6.9 Hz); 6.69 (1H, s, H-7); 6.8 (1H, s, H-10). ^{13}C-NMR δ (DMSO-d_6): 27.64 (1C, C-6); 35.68 (1C, C-2); 36.5 (1C, C-5); 43.34 (1C, C-1); 55.36 (1C, CH_3O); 55.60 (1C, CH_3O); 58.01 (1C, C-10_b); 110.12 (1C, C-7); 111.81 (1C, C-10); 125.29 (1C, C-6_a); 126.80 (1C, C-10_a); 147.08 (1C, C-8); 147.35 (1C, C-9); 171.30 (1C, C-3); 173.79 (1C, CO). HRMS: Calc. for $C_{15}H_{18}NO_5$ $[M+H]^+$ 292.1185; found 292.1184

(±)-*trans*- and *cis*-9,10-Dimethoxy-4-oxo-2,3,4,6,7,11b-hexahydro-1*H*-pyrido[2,1-*a*]isoquinoline-1-carboxylic acid (22): Obtained from reaction between 3,4-dihydroisoquinoline (**18**) and 0.160 g glutaric anhydride (**6**). Purification by means of column chromatography (ethyl acetate/cyclohexane/formic acid 5:1:0.1) followed by recrystallization (ethyl acetate) yielded the two diastereomers as white solids. Total yield is 0.251 g, 75%.

cis-**22**: Isolated 0.124 g (37%). Mp 199.0-201.0°C (ethyl acetate). IR (KBr): 3670-2400 (OH), 1739 (CO), 1635 (CON) cm^{-1}. ^{1}H-NMR δ (DMSO-d_6): 1.96-2.06 (1H, m, H-2); 2.15 (1H, ddt, H-2, *J*=5.7; 8.2; 13.9 Hz); 2.27 (1H, m, H-3); 2.3 (1H, m, H-3); 2.55 (1H, ddd, H-6, *J*=4.5; 10.2;12.6 Hz); 2.66 (2H, m, H-7); 3.54 (1H, ddd, H-6, *J*=4.5; 5.4; 12.6 Hz); 3.70 (3H, s, CH_3O); 3.71 (3H, s, CH_3O); 4.68 (1H, m, H-1, *J*=4.2 Hz); 4.84 (1H, d, H-11b, *J*=4.2 Hz); 6.68 (1H, s, H-8); 6.88 (1H, s, H-10); 12.50-13.40 (1H, br.s, COOH). ^{13}C-NMR δ (DMSO-d_6): 22.03 (1C, C-2); 27.72 (1C, C-7); 28.76 (1C, C-3); 38.28 (1C, C-6); 43.24 (1C, C-1); 55.39 (1C, CH_3O); 55.72 (1C, CH_3O); 56.50 (1C, C-11_b); 109.44 (1C, Ph); 111.62 (1C, Ph); 127.08 (1C, Ph); 127.86 (1C, Ph); 147.03 (1C, Ph); 147.24 (1C, Ph); 167.91 (1C, C-4); 173.08 (1C, COOH). HRMS: Calc. for $C_{16}H_{20}NO_5$ $[M+H]^+$ 306.1341; found 306.1345.

trans-**22:** Isolated 0.127 g (38 %). Mp 198.0-199.6°C (ethyl acetate). IR (KBr): 3670-2400 (OH), 1740 (CO), 1636 (CON) cm^{-1}. ^{1}H-NMR δ (DMSO-d_6): 1.82-1.90 (1H, m, H-2); 1.98 (1H, dtd, H-2, *J*=5.7; 8.2; 13.9 Hz); 2.22-2.36 (2H, m, H-3); 2.65 (1H, td, H-7, *J*=4.5; 15.8 Hz); 2.78 (1H, ddd, H-7, *J*=5.5; 10.2; 15.8 Hz); 2.97 (1H, ddd, H-6, *J*=4.5; 10.2;12.6 Hz); 3.10 (1H, ddd, H-1, *J*=4.2; 7.3; 8.6 Hz); 3.70 (3H, s, CH_3O); 3.73 (3H, s, CH_3O); 4.26 (1H, ddd, H-6, *J*=4.5; 5.4; 12.6 Hz); 4.92 (1H, d, H-11b, *J*=7.1 Hz); 6.77 (1H, s, H-8); 6.78 (1H, s, H-10); 12.97 (1H, br.s, COOH). ^{13}C-NMR δ (DMSO-d_6): 22.63 (1C, C-2); 27.49 (1C, C-7); 30.29 (1C, C-3); 41.03 (1C, C-6); 44.11 (1C, C-1); 55.51 (1C, CH_3O); 55.60 (1C, CH_3O); 56.67 (1C, C-11_b); 108.34 (1C, Ph); 112.22 (1C, Ph);

128.26 (1C, Ph); 128.55 (1C, Ph); 147.09 (1C, Ph); 147.72 (1C, Ph); 167.88 (1C, C-4); 175.27 (1C, COOH). HRMS: Calc. for $C_{16}H_{20}NO_5$ $[M+H]^+$ 306.1341; found 306.1352.

(±)-*trans*- and *cis*-9,10-Dimethoxy-4-oxo-1,3,4,6,7,11b-hexahydro-[1,4]ox-azino[2,1-*a*]isoquinoline-1-carboxylic acid (23): Obtained from 3,4-dihydroisoquinoline (**18**) and 0.163 g diglycolic anhydride (**7**). Purification by means of column chromatography (ethyl acetate/cyclohexane/formic acid 5:1:0.1) followed by recrystallization (ethyl acetate/light petroleum 2:1) yielded the two diastereomers as white solids. Total yield is 0.166 g, 54%.

cis-**23**: Isolated 0.091 g (27%). Mp 150.0-150.8°C (ethyl acetate: light petroleum = 2:1). IR (KBr): 3700-2400 (OH), 1739 (CO), 1634 (CON) cm^{-1} ^{1}H-NMR δ (DMSO-d_6): 2.56 (1H, m, H-7); 2.60 (1H, m, H-7); 2.72 (1H, ddd, H-6, *J*=2.95, 11.94, 14.90 Hz); 3.71 (3H, s, CH_3O); 3.72 (3H, s, CH_3O); 4.05 (1H, s, H-3); 4.30 (1H, s, H-3); 4.62 (1H, ddd, H-6, *J*=2.51; 4.25; 12.47 Hz); 4.99 (1H, d, H-1, *J*=4.2 Hz); 5.17 (1H, d, H-11_b, *J*=4.2 Hz); 6.73 (1H, s, H-8); 7.00 (1H, s, H-11); 12.83 (1H, s, COOH). ^{13}C-NMR δ (DMSO-d_6):27.52 (1C, C-7); 37.00 (1C, C-6); 55.37 (1C, CH_3O); 55.72 (1C, CH_3O); 55.78 (1C, C-11_b); 64.45 (1C, C-3); 74.23 (1C, C-1); 110.23 (1C, C-11); 111.74 (1C, C-8); 112.55 (1C, C-7_a); 127.52 (1C, C-11_a); 147.34 (1C, C-9); 147.51 (1C, C-10); 165.54 (1C, C-4); 169.94 (1C, COOH). HRMS: Calc. for $C_{15}H_{18}NO_6$ $[M+H]^+$ 308.1134; found 308.1121.

trans-**23**: Isolated 0.091 g (27%). Mp 148.0-149.4°C (ethyl acetate: light petroleum = 2:1). IR (KBr): 3679-2346 (OH), 1739 (CO), 1621 (CON) cm^{-1}. ^{1}H-NMR δ (DMSO-d_6): 2.66 (1H, ddd, H-7, *J*=2.8; 4.0; 15.8 Hz); 2.81 (1H, ddd, H-7, *J*=5.5; 11.2; 15.8 Hz); 2.92 (1H, ddd, H-6, *J*=4.1; 11.2; 12.4 Hz); 3.69 (3H, d, CH_3O); 3.73 (3H, d, CH_3O); 4.08 (1H, d, H-3, *J*=16.4 Hz); 4.32 (1H, d, H-3, *J*=16.3 Hz); 4.45 (1H, ddd, H-6, *J*=2.8; 5.4; 12.4 Hz); 4.73 (1H, d, H-11_b, *J*=5.7 Hz); 5.03 (1H, d, H-1, *J*=5.7 Hz); 6.79 (1H, s, H-8);

6.81 (1H, s, H-11); 13.71 (1H, br.s, COOH). ^{13}C-NMR δ (DMSO-d_6): 27.55 (1C, C-7); 39.69 (1C, C-6); 55.50 (1C, CH_3O); 55.59 (1C, CH_3O); 55.81 (1C, C-11_b); 65.31 (1C, C-3); 75.50 (1C, C-1); 108.61 (1C, C-11); 112.53 (1C, C-8); 124.80 (1C, C-7_a); 128.11 (1C, C-11_a); 147.14 (1C, C-9); 147.95 (1C, C-10); 165.54 (1C, C-4); 171.04 (1C, COOH). HRMS: Calc. for $C_{15}H_{18}NO_6$ $[M+H]^+$ 308.1134; found 308.1144.

(±)-*trans*- and *cis*-9,10-Dimethoxy-4-oxo-1,3,4,6,7,11b-hexahydro-[1,4]thiazino[3,4-*a*]isoquinoline-1-carboxylic acid (24): Obtained from 3,4-dihydroisoquinoline (**18**) and 0.185 g thiodiacetic anhydride (**8**). Purification by means of column chromatography (ethyl acetate/toluene/light petroleum/formic acid 2:1:1:0.1) followed by recrystallization (ethyl acetate/acetonitrile 2:1) yielded the two diastereomers as white solids. Total yield is 0.270 g, 76%.

cis-**24**: Isolated 0.135 g (38%). Mp 205.0-206.1°C (ethyl acetate: acetonitrile = 2:1). IR (KBr): 3580-2350 (OH), 1740 (CO), 1622 (CON) cm^{-1}. ^{1}H-NMR δ (DMSO-d_6): 2.59 (1H, ddd, H-7, *J*=4.5; 4.5; 15.4 Hz); 2.77 (1H, ddd, H-7, *J*=4.5; 9.7; 15.4 Hz); 3.16 (1H, ddd, H-6, *J*=4.5; 9.7; 12.5 Hz); 3.25 (1H, H-3, *J*=15.3 Hz); 3.64 (1H, d, H-3, *J*=15.3 Hz); 3.72 (3H, s, CH_3O); 3.73 (3H, s, CH_3O); 4.24 (1H, ddd, H-6, *J*=4.5; 4.5; 12.6 Hz); 4.54 (1H, d, H-1, *J*=4 Hz); 5.08 (1H, d, H-11_b, *J*=4 Hz); 6.74 (1H, s, H-8); 7.04 (1H, s, H-11); 12.45 (1H, br.s., COOH). ^{13}C-NMR δ (DMSO-d_6): 27.37 (1C, C-7); 28.05 (1C, C-3); 38.53 (1C, C-6); 44.19 (1C, C-1); 55.41 (1C, CH_3O); 55.79 (1C, CH_3O); 57.04 (1C, C-11_b); 110.17 (1C, C-8); 111.57 (1C, C-11); 124.75 (1C, C-7_a); 127.74 (1C, C-11_a); 147.41 (1C, C-9); 147.61 (1C, C-10); 166.12 (1C, C-4); 170.86 (1C, COOH). HRMS: Calc. for $C_{15}H_{18}NO_5S$ $[M+H]^+$ 324.0906; found 324.0903.

trans-**24**: Isolated 0.135 g (38%). Mp 205.7-207.0°C (ethyl acetate: acetonitrile = 2:1). IR (KBr): 3580-2350 (OH), 1741 (CO), 1620 (CON) cm^{-1}. ^{1}H-NMR δ (DMSO-d_6): 2.74 (1H, m, H-6); 2.76 (1H, m, H-7); 2.80 (1H, m, H-7); 3.11 (1H, d, H-3, *J*=14.5 Hz); 3.55

(1H, d, H-1, *J*=10.3 Hz); 3.69 (3H, s, CH_3O); 3.76 (3H, s, CH_3O); 3.90 (1H, d, H-3, *J*=14.5 Hz); 4.34 (1H, ddd, H-6, *J*=2.3; 4.2; 12.1 Hz); 5.05 (1H, d, H-11_b, *J*=10.3 Hz); 6.80 (1H, s, H-8), 6.90 (1H, s, H-11); 12.75 (1H, br. s., COOH). ^{13}C-NMR δ (DMSO-d_6): 27.50 (1C, C-7); 29.49 (1C, C-3); 38.86 (1C, C-6); 40.20 (1C, C-1); 55.33 (1C, CH_3O); 55.40 (1C, CH_3O); 55.98 (1C, C-11_b); 110.84 (1C, C-8); 112.37 (1C, C-11); 124.15 (1C, C-7_a); 127.86 (1C, C-11_a); 146.80 (1C, C-9); 148.31 (1C, C-10); 169.15 (1C, C-4); 172.69 (1C, COOH). HRMS: Calc. for $C_{15}H_{18}NO_5S$ $[M+H]^+$ 324.0906; found 324.0906.

2-(6,7-Dimethoxy-3,4-dihydroisoquinolin-1(2*H*)-ylidene)cyclohexane-1,3-dione (25): Obtained from 0.205 g 3,4-dihydroisoquinoline (**19**) and 0.171 g glutaric anhydride (**6**). Purification by means of column chromatography (ethyl acetate/2-propanol 4:1) followed by recrystallization from methanol yielded 0.201 g of **25** as off-white solid. Total yield is 70%. Mp 239.1–240.4°C (ethyl acetate). IR (KBr) 3432 (NH), 1628 (CO), 1593 (C=C) cm^{-1}. ^{1}H-NMR δ (DMSO-d_6):1.89 (2H, d, H-5, *J*=6.55 Hz); 2.35 (4H, t, H-4, H-6, *J*=6.3 Hz); 2.77 (2H, t, H-4’, *J*=6.9 Hz); 3.46 (2H, m, H-3’); 3.65 (3H, s, CH_3O); 3.84 (3H, s, CH_3O); 6.93 (1H, s, H-6’); 6.95 (1H, s, H-8’); 12.52 (1H, s, NH). ^{13}C-NMR δ (DMSO-d_6): 19.33 (1C, C-5); 26.85 (1C, C-4’); 37.99 (2C, C-4, C-6); 38.35 (1C, C-3’); 55.65 (1C, CH_3O); 55.73 (1C, CH_3O); 105.98 (1C, C-2); 110.31 (1C, C-5’); 115.40 (1C, C-8’); 119.26 (1C, C-4a’); 131.98 (1C, C-8a’); 146.09 (1C, C-6a’); 152.57 (1C, C-7a’); 165.76 (1C, C-1’); 195.19 (2C, C-1, C-3). HRMS: Calc. for $C_{17}H_{20}NO_4$ $[M+H]^+$ 302.1392; found 302.1402.

4-(6,7-Dimethoxy-3,4-dihydroisoquinolin-1(2*H*)-ylidene)-2*H*-pyran-3,5(4*H*,6*H*)-dione (26): Obtained from 0.205 g 3,4-dihydroisoquinoline (**19**) and 0.174 g diglycolic anhydride (**7**). Purification by means of column chromatography (ethyl acetate/2-propanol 4:1) followed by recrystallization from methanol yielded 0.188 g of **26** as yellowish solid. Total yield is 62%. Mp 235.8–236.3°C (methanol). IR (KBr) 3447 (NH), 1640 (CO), 1590 (C=C) cm^{-1}. ^{1}H-NMR δ (DMSO-d_6): 2.81 (2H, t, H-4', *J*=6.95 Hz); 3.53 (2H, m, H-3'); 3.67 (3H, s, CH_3O); 3.86 (3H, s, CH_3O); 4.08 (4H, s, H-2, H-4); 6.97 (1H,s, H-5'); 7.04 (1H, s, H-8'); 12.27 (1H, s, NH). ^{13}C-NMR δ (DMSO-d_6): 25.71 (1C, C-4'); 38.59 (1C, C-3'); 55.68 (1C, CH_3O); 55.83 (1C, CH_3O); 71.97 (1C, C-6); 78.18 (1C, C-2); 103.08 (1C, C-4); 110.45 (1C, C-5'); 115.29 (1C, C-8'); 118.24 (1C, C-4a'); 132.53 (1C, C-8a'); 146.24 (1C, C-6'); 153.20 (1C, C-7'); 164.95 (1C, C-1'); 191.93 (2C, C-3, C-5). HRMS: Calc. for $C_{16}H_{18}NO_5$ $[M+H]^+$ 304.1185; found 304.1176.

5-(6,7-Dimethoxy-3,4-dihydroisoquinolin-1(2*H*)-ylidene)-4-oxopentanoic acid (27): Obtained from 3,4-dihydroisoquinoline (**19**) and 0.150 g succinic anhydride (**5**). Purification by means of column chromatography (ethyl acetate/2-propanol 8:1) followed by recrystallization form ethyl acetate/methanol yielded 0.134 g of **27** as white solid. Yield is 44 %. Mp 241.5-243°C (ethyl acetate/methanol). IR (KBr) 3422 (NH), 3100-2500 (OH), 1719 (CO), 1644 (C=C). ^{1}H-NMR *δ* (DMSO-d_6): 2.45 (2H, t, H-2, *J*=6.9 Hz); 2.57 (2H, t, H-3, *J*=6.8 Hz); 2.79 (2H, t, H-4', *J*=6.51 Hz); 3.38 (2H, m, H-3'); 3.81 (3H, s, CH_3O); 3.82 (3H, s, CH_3O); 5.70 (1H, s, H-5); 6.91 (1H, s, H-5'); 7.24 (1H, s, H-8'); 10.90 (1H, s, NH); 12.03 (1H, br. s., COOH). ^{13}C-NMR δ (DMSO-d_6): 27.25 (1C, C-4'); 29.34 (1C, C-2); 36.05 (1C, C-3); 38.08 (1C, C-3'); 55.62 (1C, CH_3O); 55.85 (1C, CH_3O); 88.11 (1C, C-5); 108.69 (1C, C-5'); 111.35 (1C, C-8'); 120.35 (1C, C-4a'); 130.65 (1C, C-8a'); 147.56 (1C, C-6'); 151.32 (1C, C-7'); 156.02 (1C, C-1'); 174.25 (1C, C-1). HRMS: Calc. for $C_{16}H_{20}NO_5$ $[M+H]^+$ 306.134149; found 306.13439.

(±)-*trans*-9,10-Dimethoxy-11b-methyl-4-oxo-1,3,4,6,7,11b-hexahydro-[1,4]thiazino[3,4-*a*]isoquinoline-1-carboxylic acid (28): Obtained from 1-methyl-3,4-dihydroisoquinoline (**19**) and 0.198 g thiodiacetic anhydride (**8**). Purification by means of column chromatography (ethyl acetate/cyclohexane/formic acid 3:2:0.1) followed by recrystallization form ethyl acetate yielded 0.239 g of **28** as white solid. Yield is 71%. Mp 207.1-208.5°C (ethyl acetate). IR (KBr) 3500-2400 (OH), 1724 (COOH), 1613 (CON) cm^{-1}. ^{1}H-NMR *δ* (DMSO-d_6):1.82 (3H, s, 11_b-CH_3); 2.53 (1H, m, H-6); 2.59 (1H, ddd, H-6, *J*=4.3 Hz, 13.7 Hz); 2.76 (1H, ddd, H-5, *J*=3.4 Hz; 12.4 Hz); 3.28 (1H, d, H-3, *J*=17.3 Hz); 3.44 (1H, d, H-3, *J*=17.3 Hz); 3.71 (3H, s, CH_3O); 3.72 (3H, s, CH_3O); 4.33 (1H, s, H-1); 4.80 (1H, m, H-6); 6.65 (1H, s, H-8); 7.05 (1H, s, H-11); 12.34 (1H, s, COOH). ^{13}C-NMR δ (DMSO-d_6): 26.16 (1C, C-3); 28.03 (1C, C-11_b-CH3); 28.29 (1C, C-7); 36.49 (1C, C-6); 45.79 (1C, C-1); 55.31 (1C, CH_3O); 56.04 (1C, CH_3O); 61.67 (1C, C-11_b); 110.04 (1C, C-11); 111.43 (1C, C-8); 128.01 (1C, C-7_a); 130.88 (1C, C-11_a); 147.28 (1C, C-9); 147.3 (1C, C-10); 163.06 (1C, C-4); 169.89 (1C, COOH). HRMS: Calc. for $C_{16}H_{20}NO_5S$ $[M+H]^+$ 338.1062; found 338.1051.

(±)-*trans*-9,10-Dimethoxy-11b-ethyl-4-oxo-1,3,4,6,7,11b-hexahydro-[1,4]thiazino[3,4-*a*]isoquinoline-1-carboxylic acid (29): Obtained from 1-ethyl-3,4-dihydroisoquinoline (**20**) and 0.198 g thiodiacetic anhydride (**8**). Purification by means of column chromatography (ethyl acetate/cyclohexane/formic acid 3:2:0.1) followed by recrystallization form ethyl acetate yielded 0.105 g of **29** as off-white solid. Yield is 30%. Mp 146.2-147.8°C (ethyl acetate). IR (KBr) 3500-2400 (OH), 1724 (COOH), 1613 (CON) cm^{-1}. ^{1}H-NMR *δ* (DMSO-d_6): 0.84 (3H, t, CH_3, *J*=7.4 Hz); 2.23 (1H, ddd, CH_2-11_b, *J*=7.6, 15.2 Hz); 2.45 (1H, m, CH_2-11_b); 2.61 (2H, m, H-7); 2.79 (1H, ddd, H-6, *J*=3.7, 12.5 Hz); 3.41 (1H, d, H-3, *J*=16.3 Hz); 3.44 (1H, d, H-3, *J*=16.9 Hz); 3.72 (3H, s, CH_3O); 3.73 (3H, s, CH_3O); 4.17 (1H, s, H-1); 4.87 (1H, ddd, H-6, *J*=2.5, 3.7, 12.8

Hz); 6.68 (1H, s, H-8); 6.99 (1H, s, H-11); 12.26 (1H, s, COOH). ^{13}C-NMR δ (DMSO-d_6):9.75 (1C, CH_3); 25.74 (1C, C-3); 27.72 (1C, C-7); 34.87 (1C, R-CH_2-11_b); 38.24 (1C, C-6); 46.36 (1C, C-1); 55.27 (1C, CH_3O); 56.01 (1C, CH_3O); 63.93 (1C, C-11b); 110.22 (1C, C-11); 111.32 (1C, C-8); 128.40 (1C, C-7a); 129.03 (1C, C-11a); 147.34 (1C, C-9); 147.45 (1C, C-10); 163.92(1C, C-4); 170.55 (1C, COOH). HRMS: Calc. for $C_{17}H_{22}NO_5S$ $[M+H]^+$ 352.1219; found 352.1218.

^{1}H NMR spectrum of (±)-*cis*-**21** (only one enantiomer is shown)

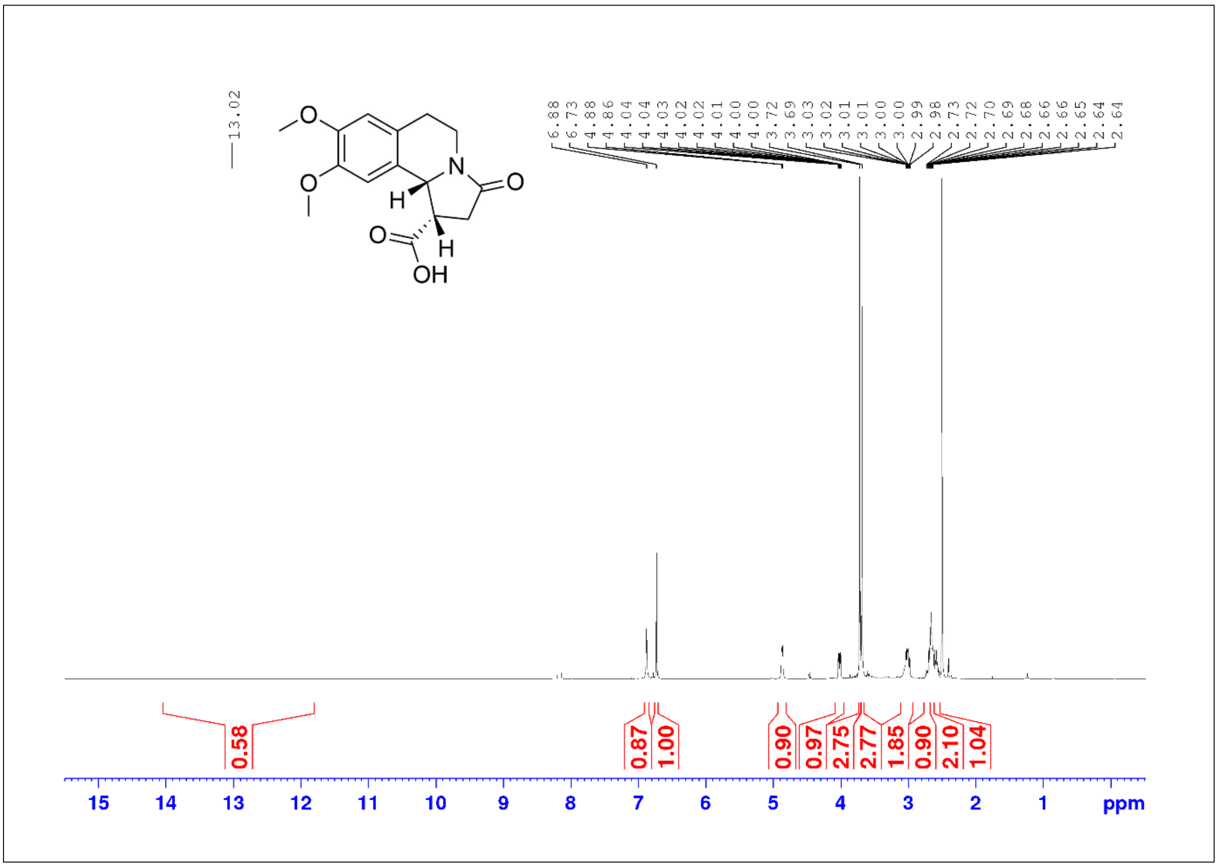

^{13}C NMR spectrum of (±)-*cis*-**21**

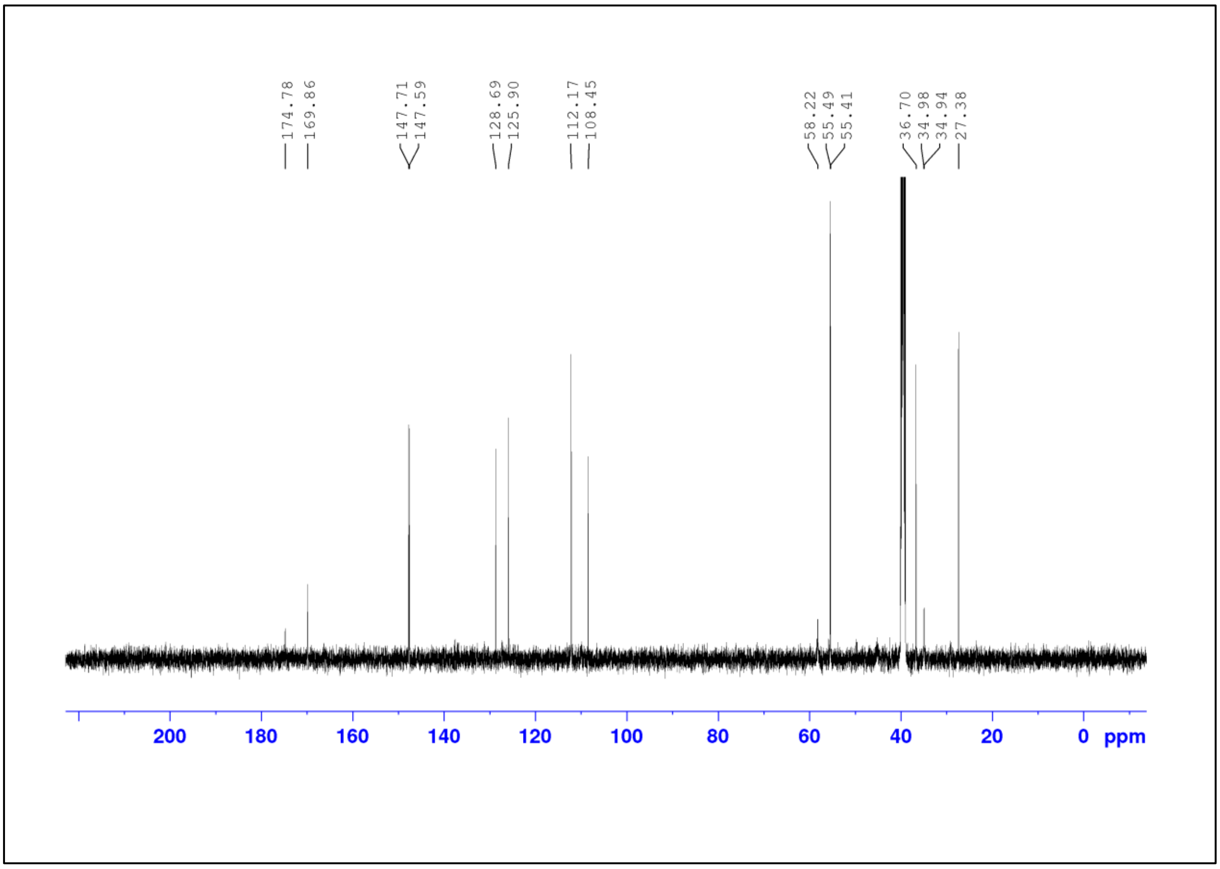

¹H NMR spectrum of (±)-*trans*-**21** (only one enantiomer is shown)

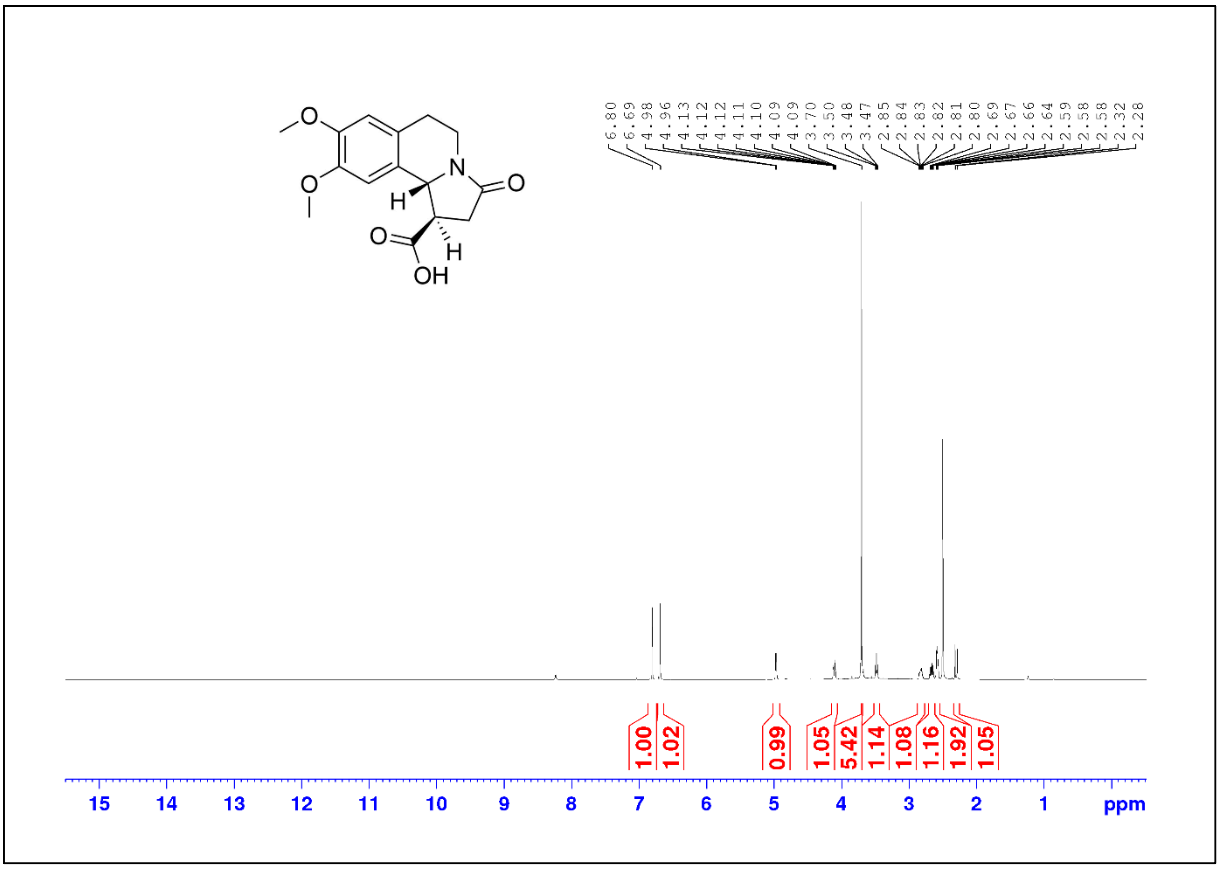

¹³C NMR spectrum of (±)-*trans*-**21**

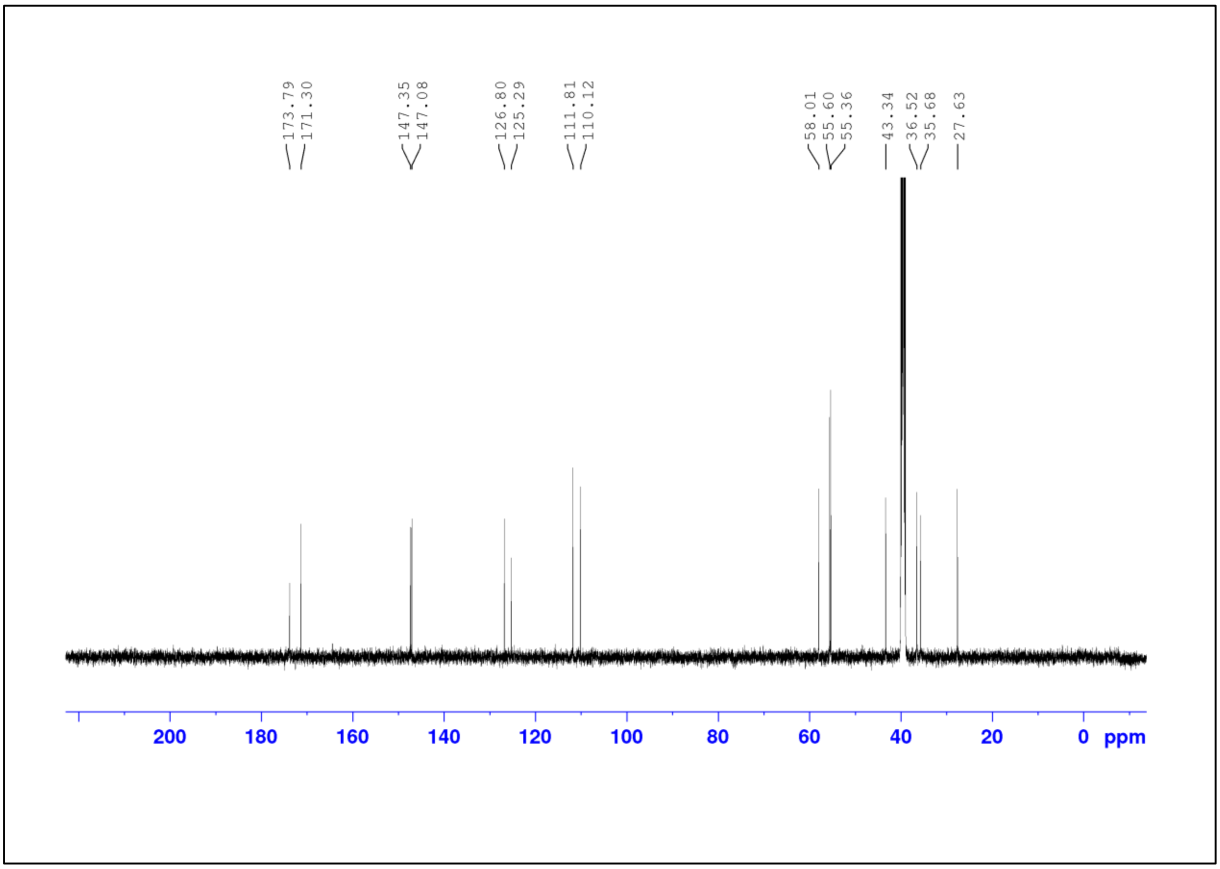

^{1}H NMR spectrum of (±)-*cis*-**22** (only one enantiomer is shown)

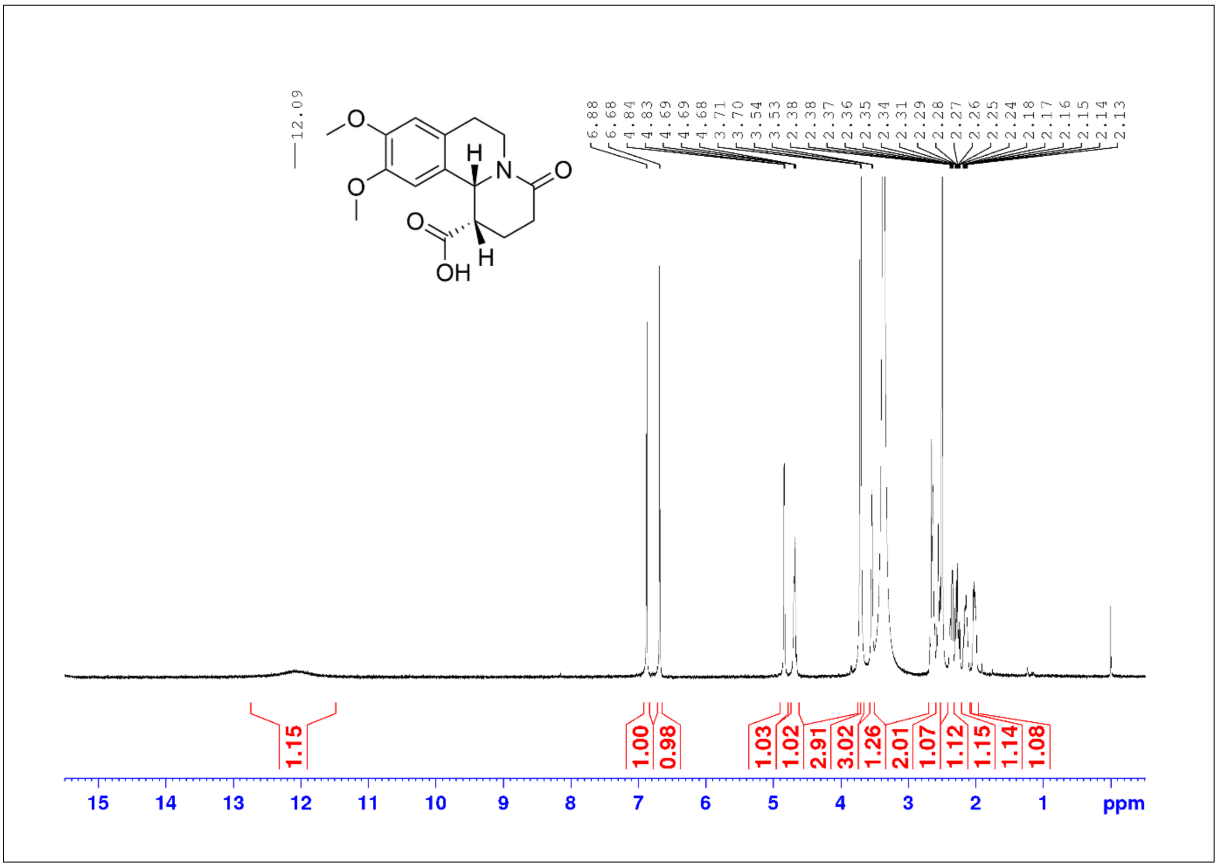

^{13}C NMR spectrum of (±)-*cis*-**22**

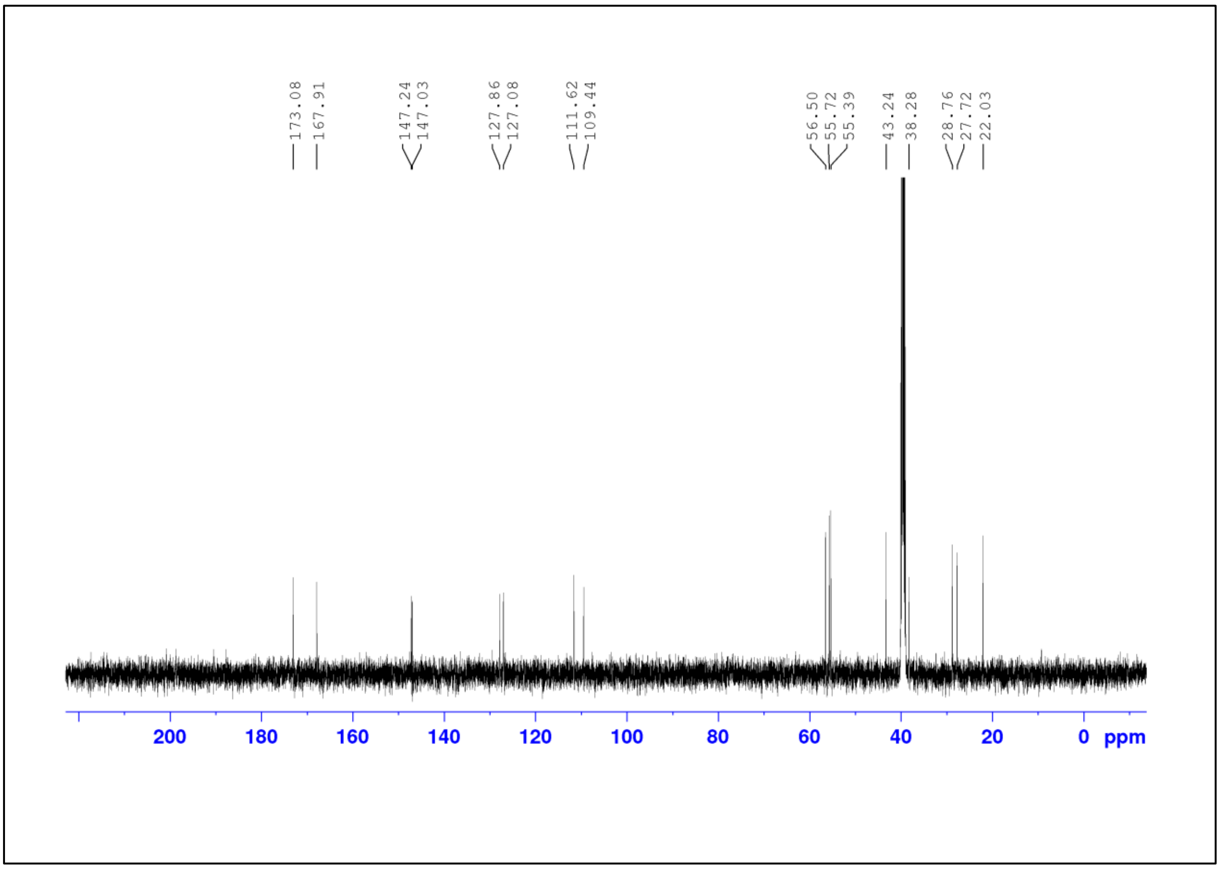

^{1}H NMR spectrum of (±)-*trans*-**22** (only one enantiomer is shown)

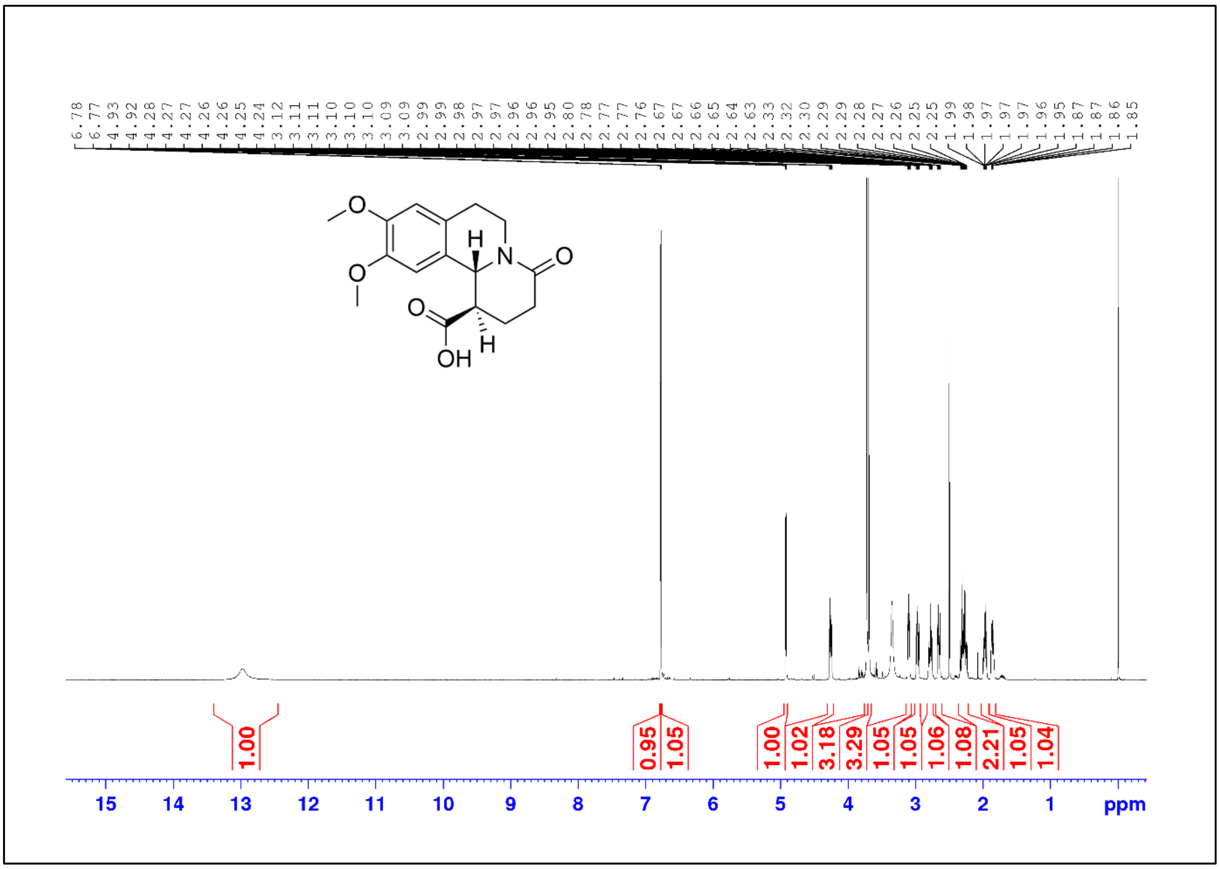

^{13}C NMR spectrum of (±)-*trans*-**22**

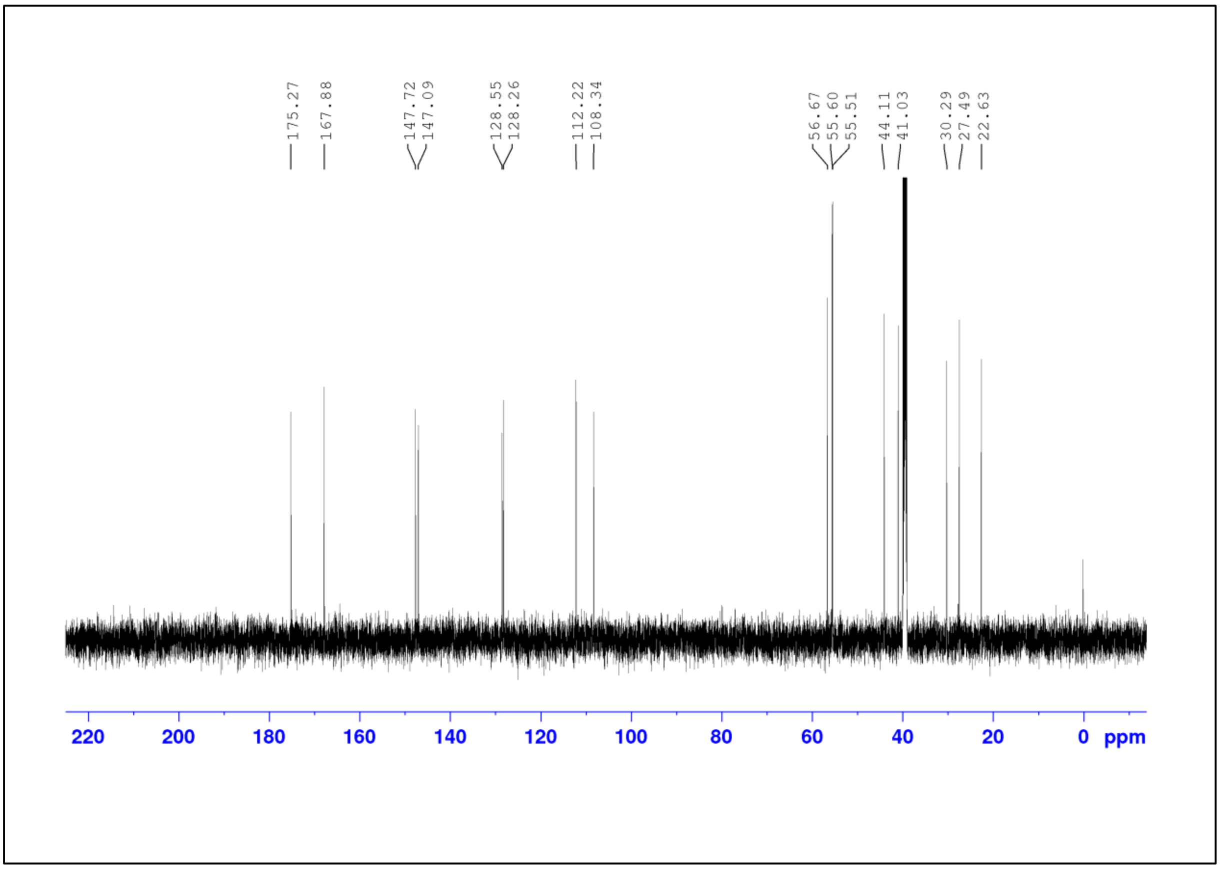

^{1}H NMR spectrum of (±)-*cis*-**23** (only one enantiomer is shown)

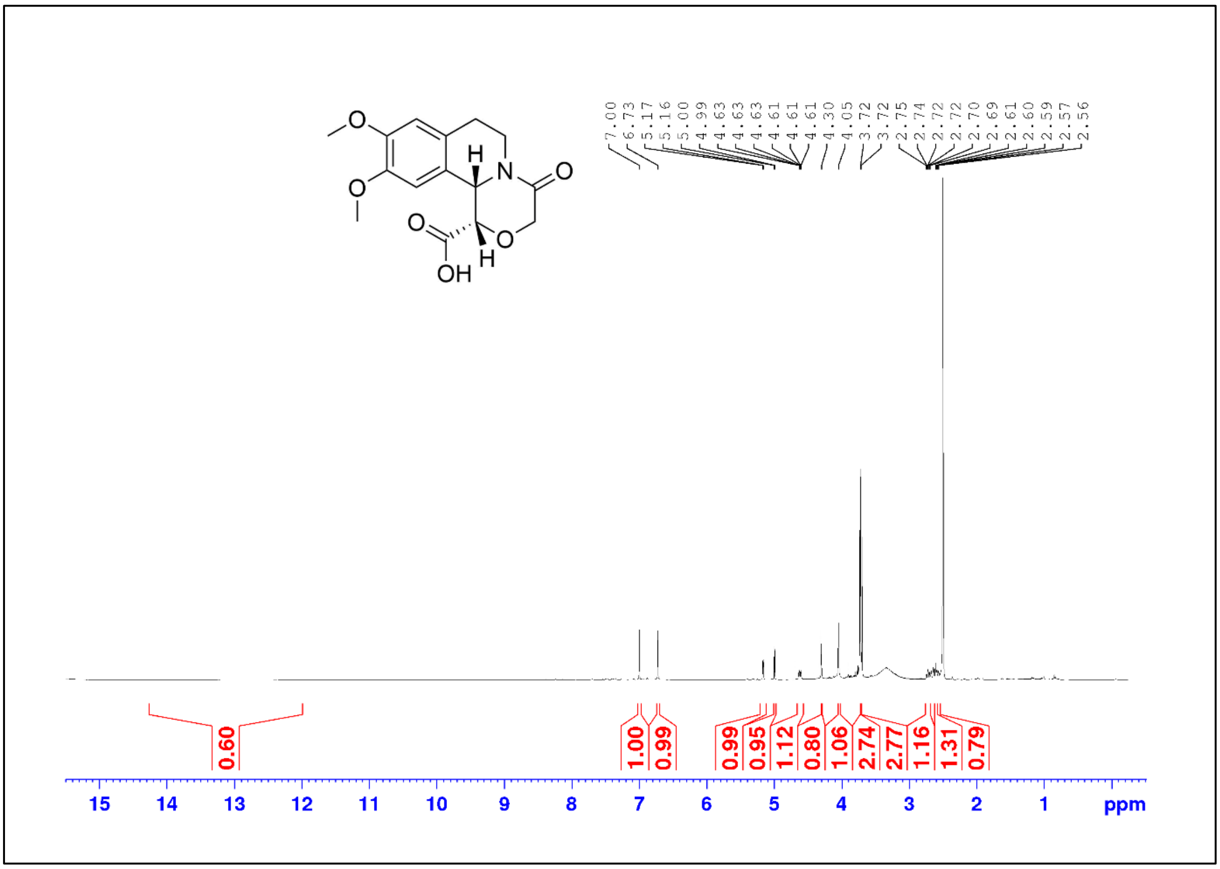

^{13}C NMR spectrum of (±)-*cis*-**23**

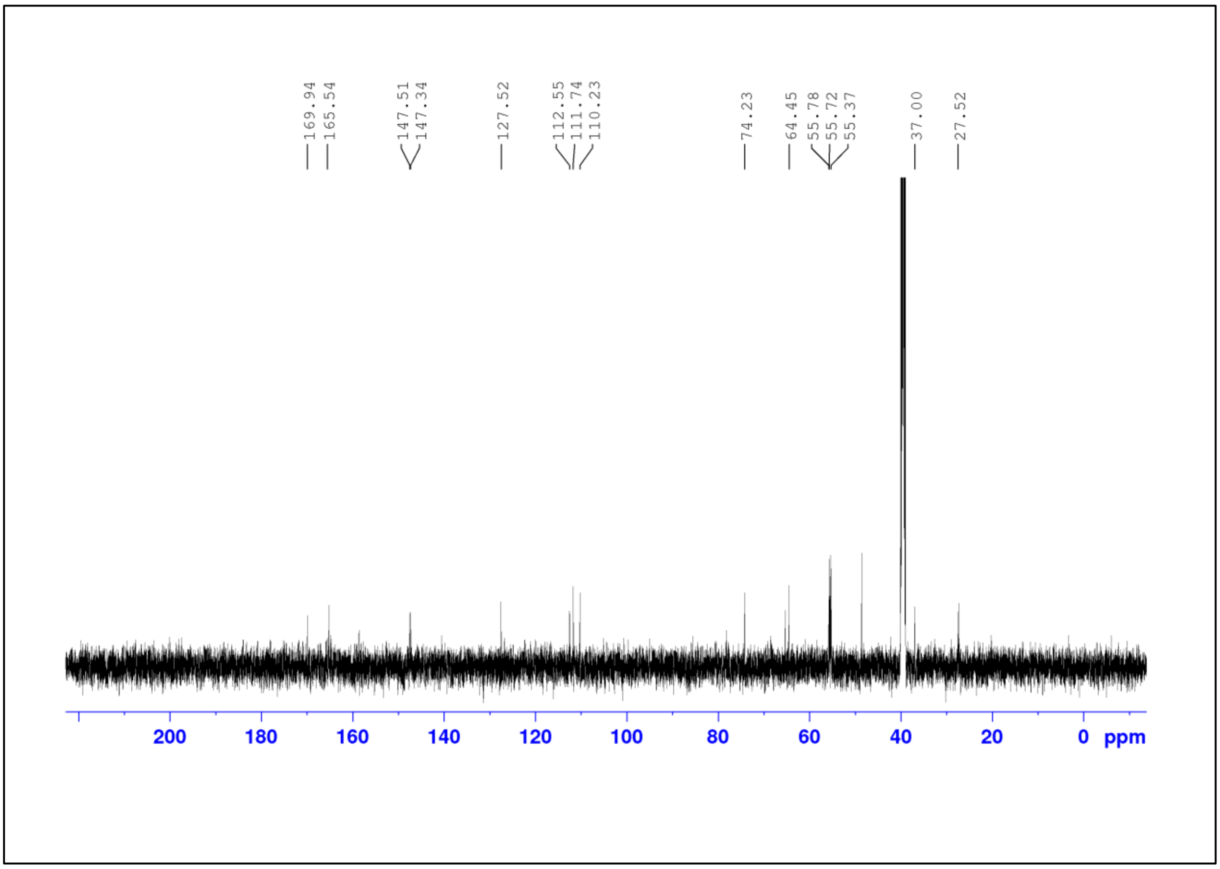

^{1}H NMR spectrum of (±)-*trans*-**23** (only one enantiomer is shown)

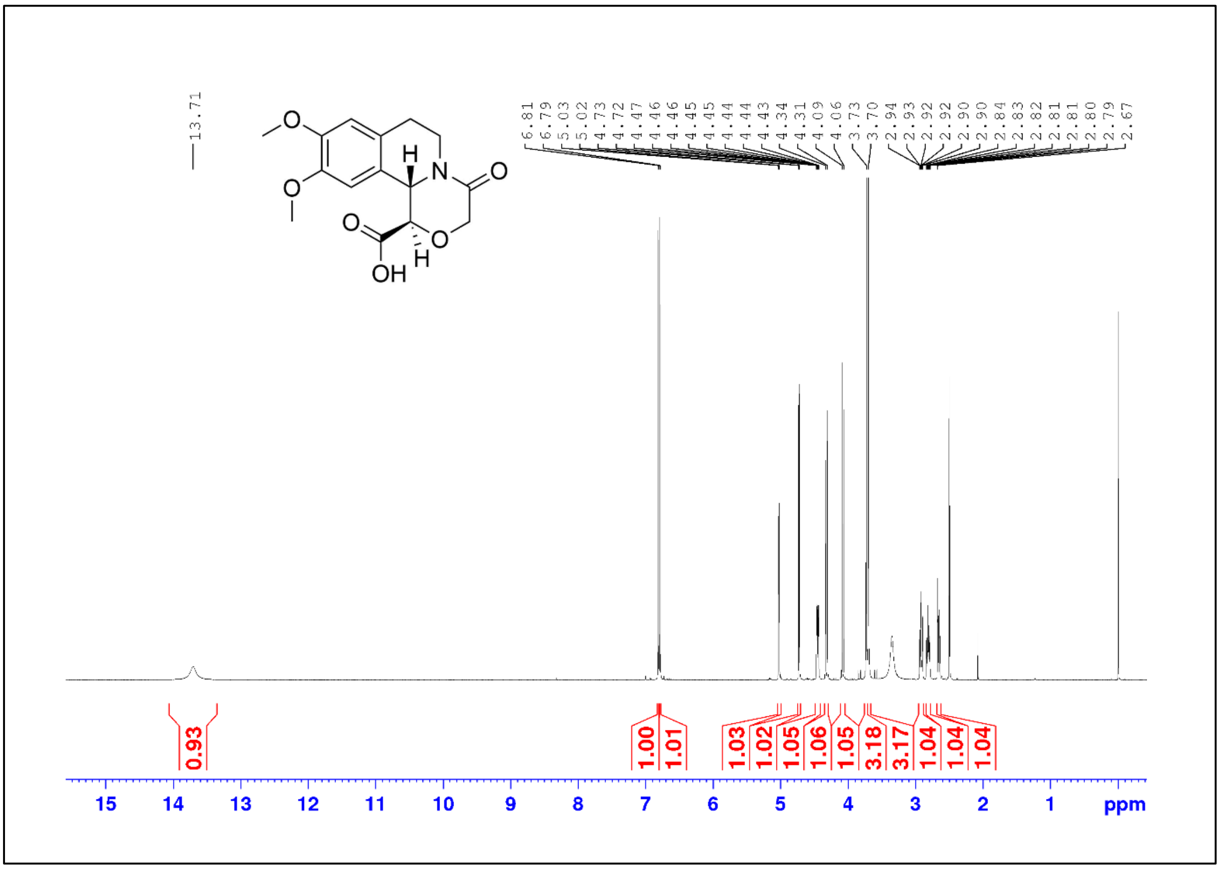

^{13}C NMR spectrum of (±)-*trans*-**23**

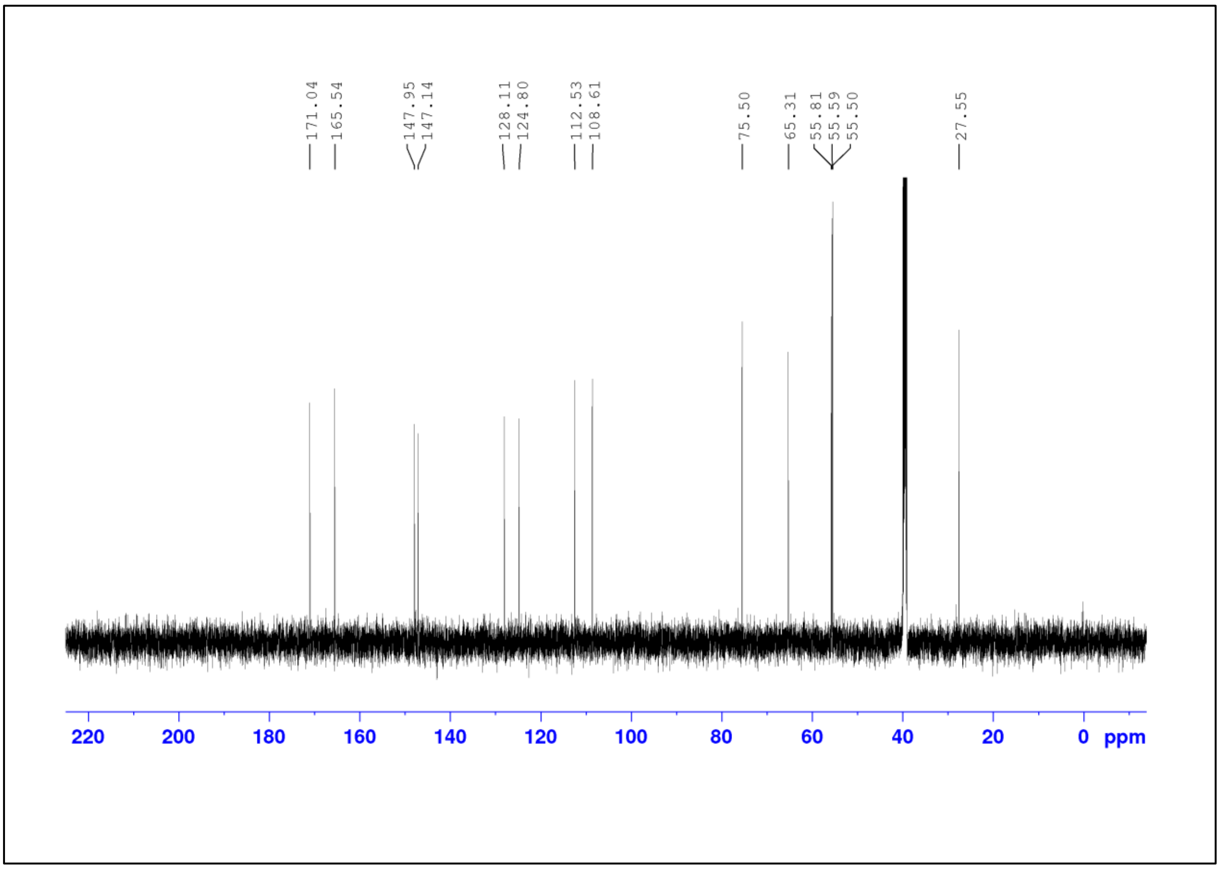

^{1}H NMR spectrum of (±)-*cis*-**24** (only one enantiomer is shown)

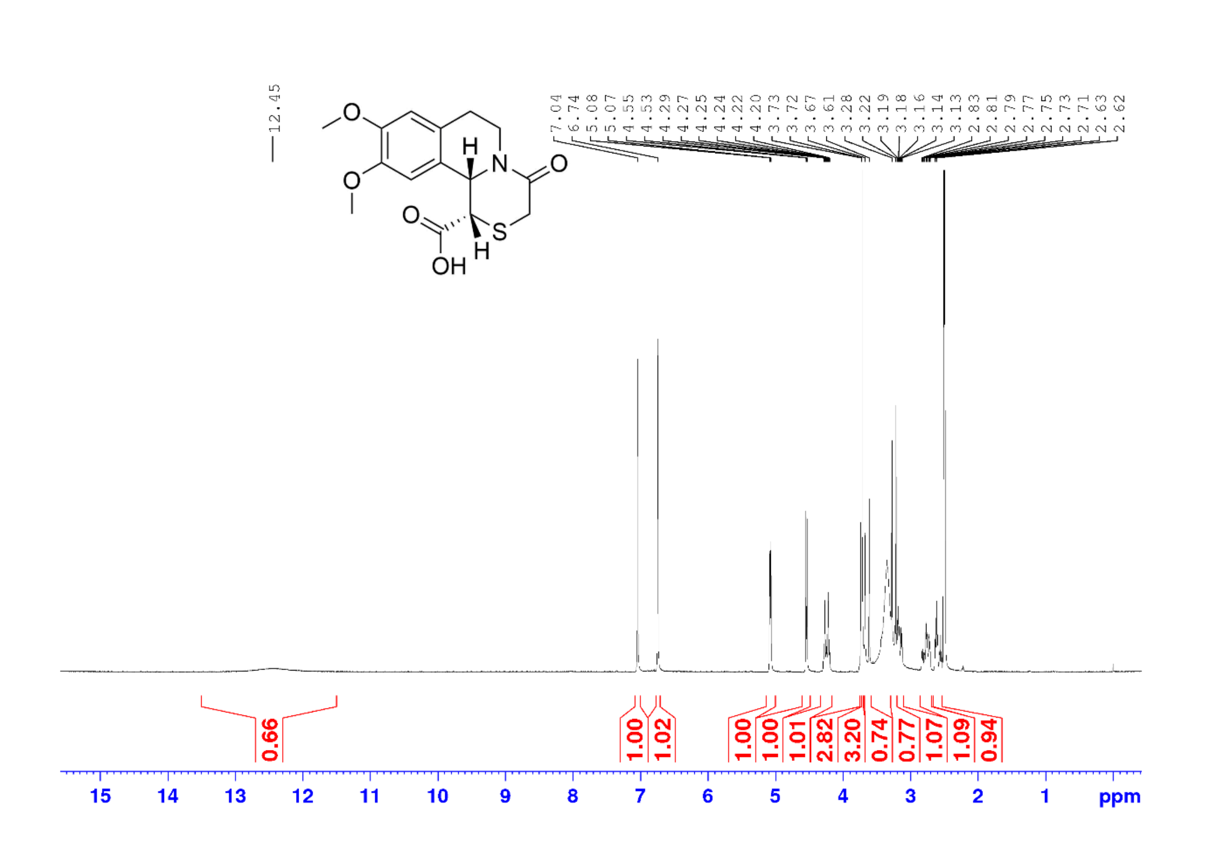

^{13}C NMR spectrum of (±)-*cis*-**24**

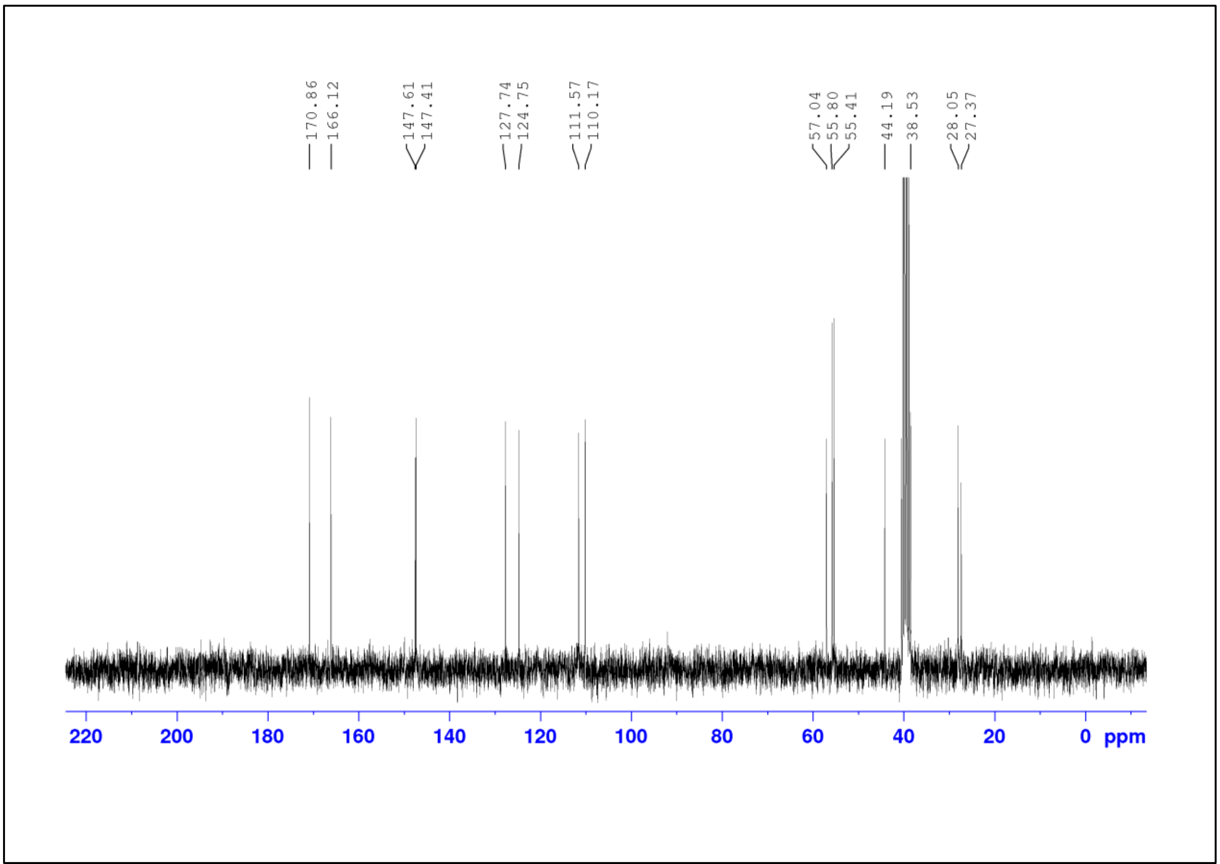

^{1}H NMR spectrum of (±)-*trans*-**24** (only one enantiomer is shown)

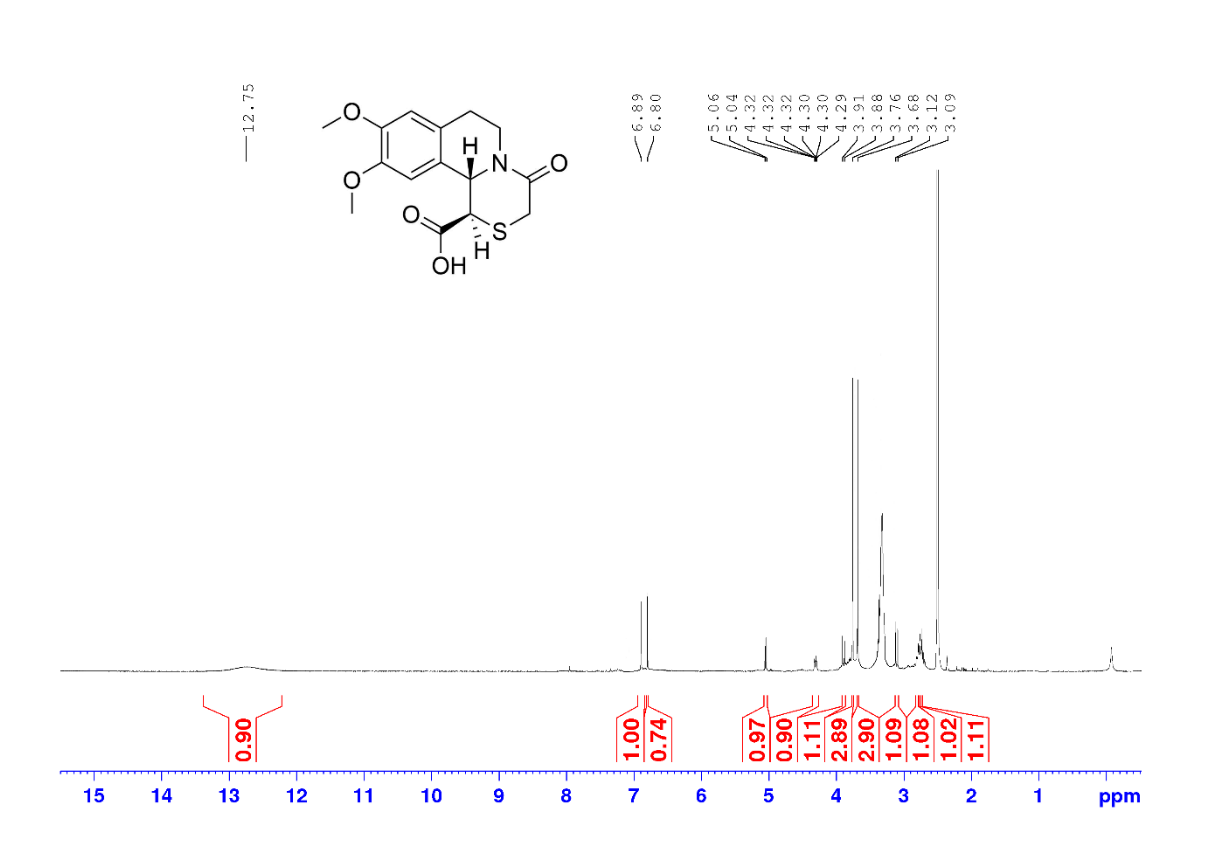

^{13}C NMR spectrum of (±)-*trans*-**24**

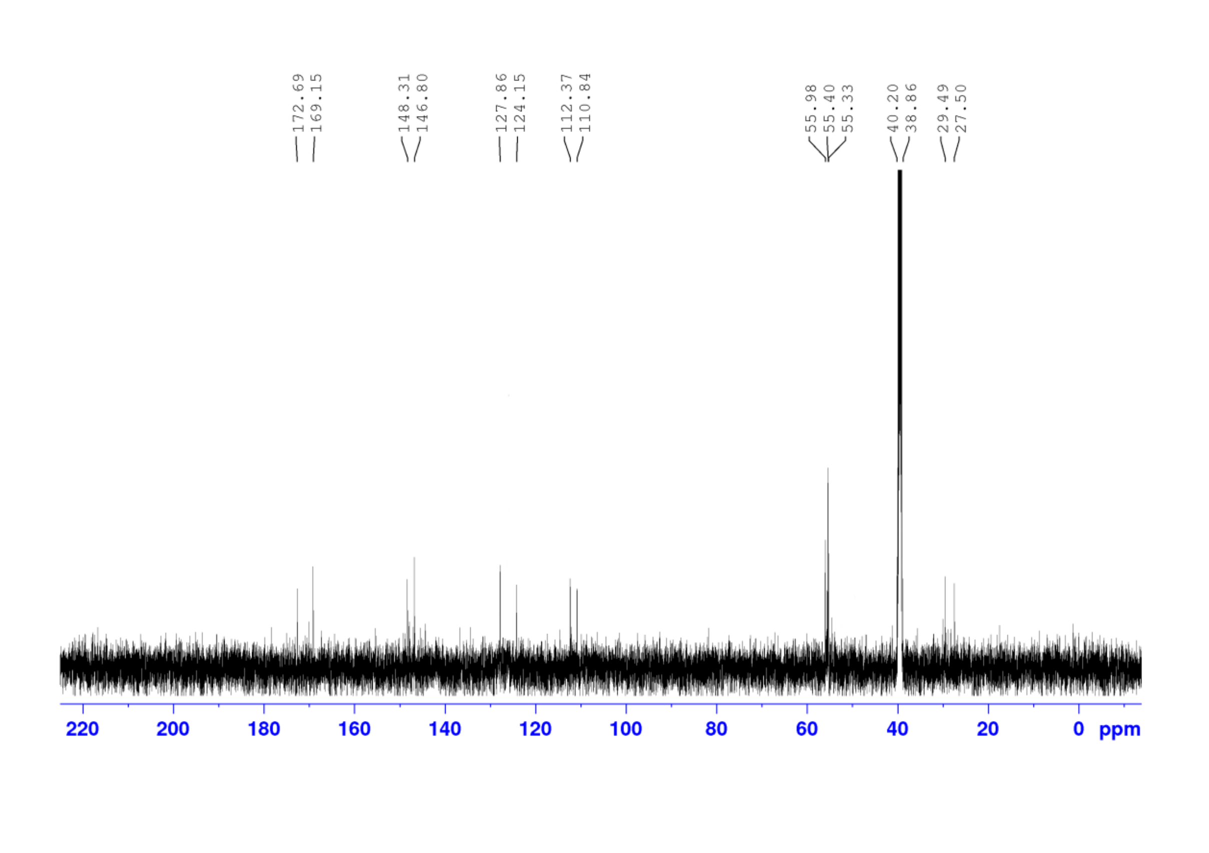

^{1}H NMR spectrum of **25**

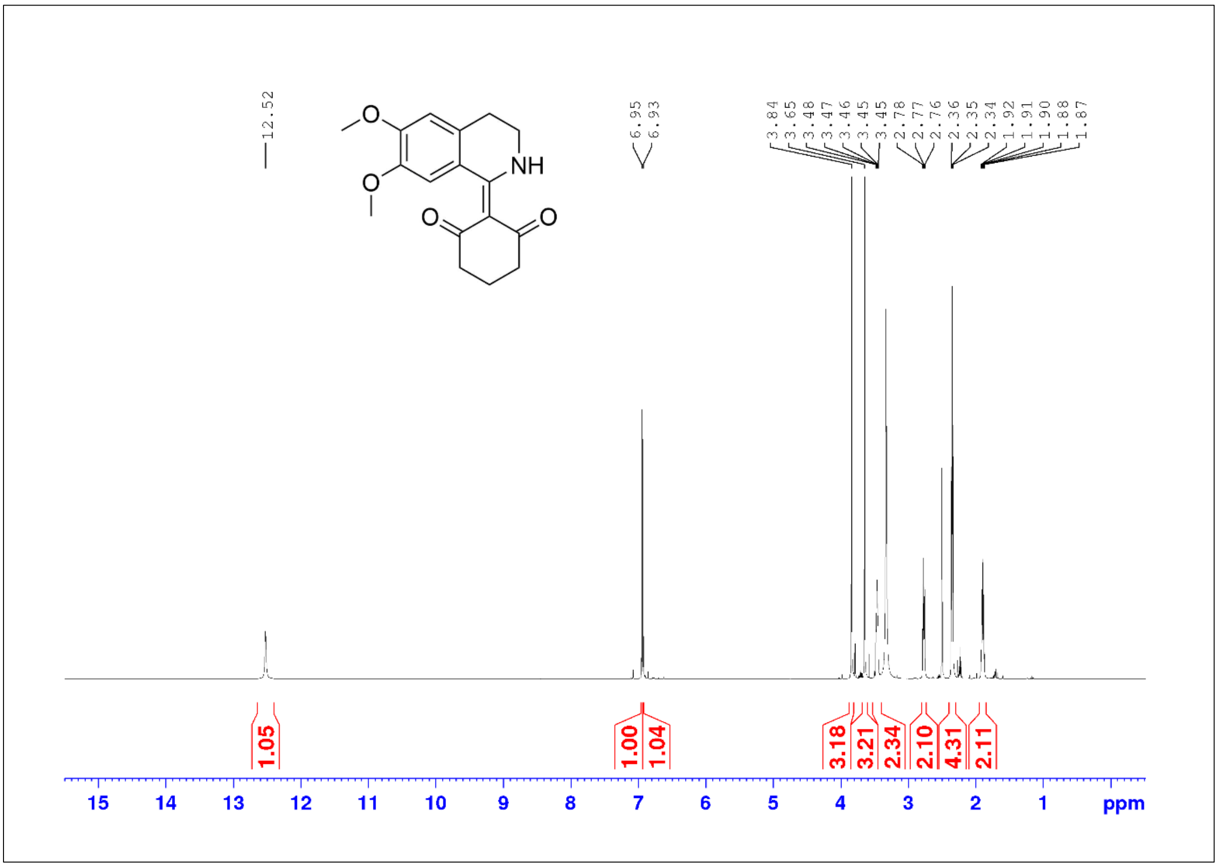

^{13}C NMR spectrum of **25**

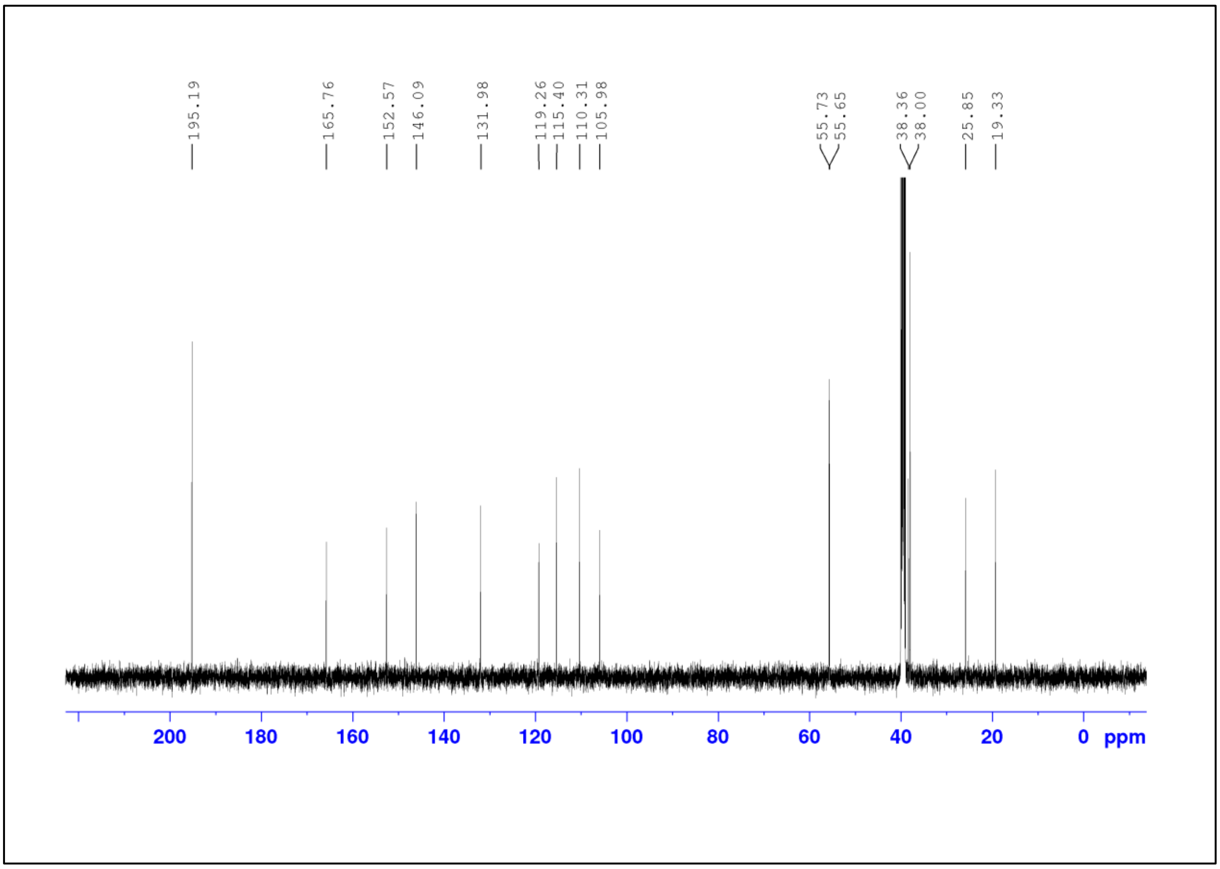

^{1}H NMR spectrum of **26**

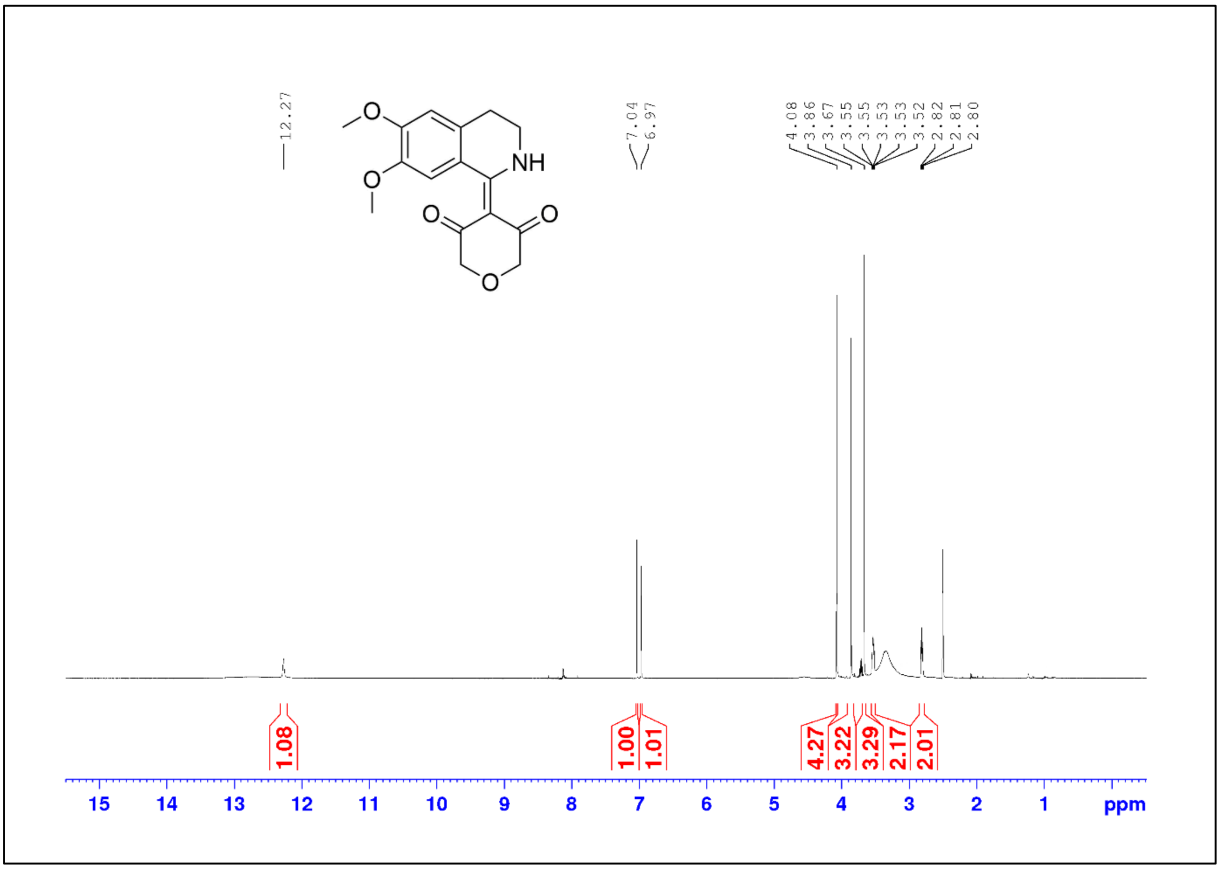

^{13}C NMR spectrum of **26**

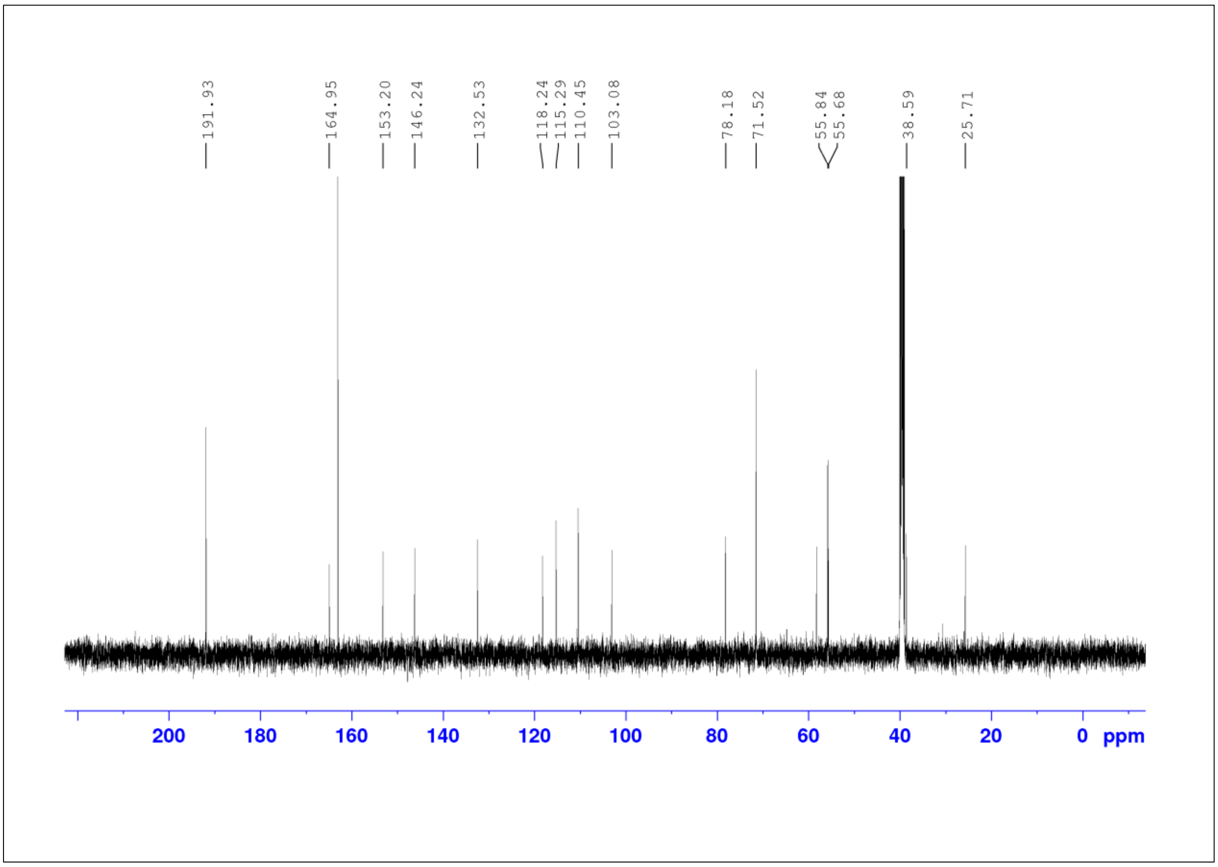

^{1}H NMR spectrum of **27**

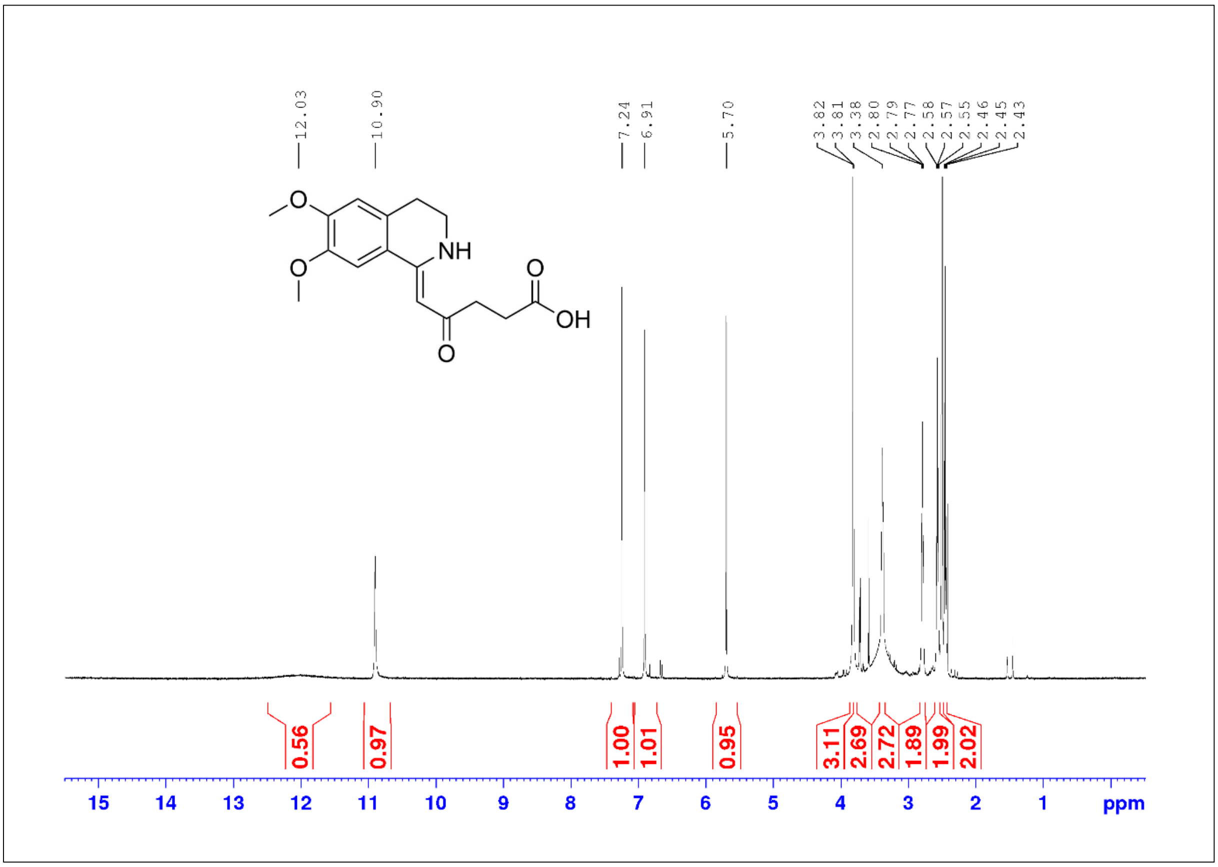

^{13}C NMR spectrum of **27**

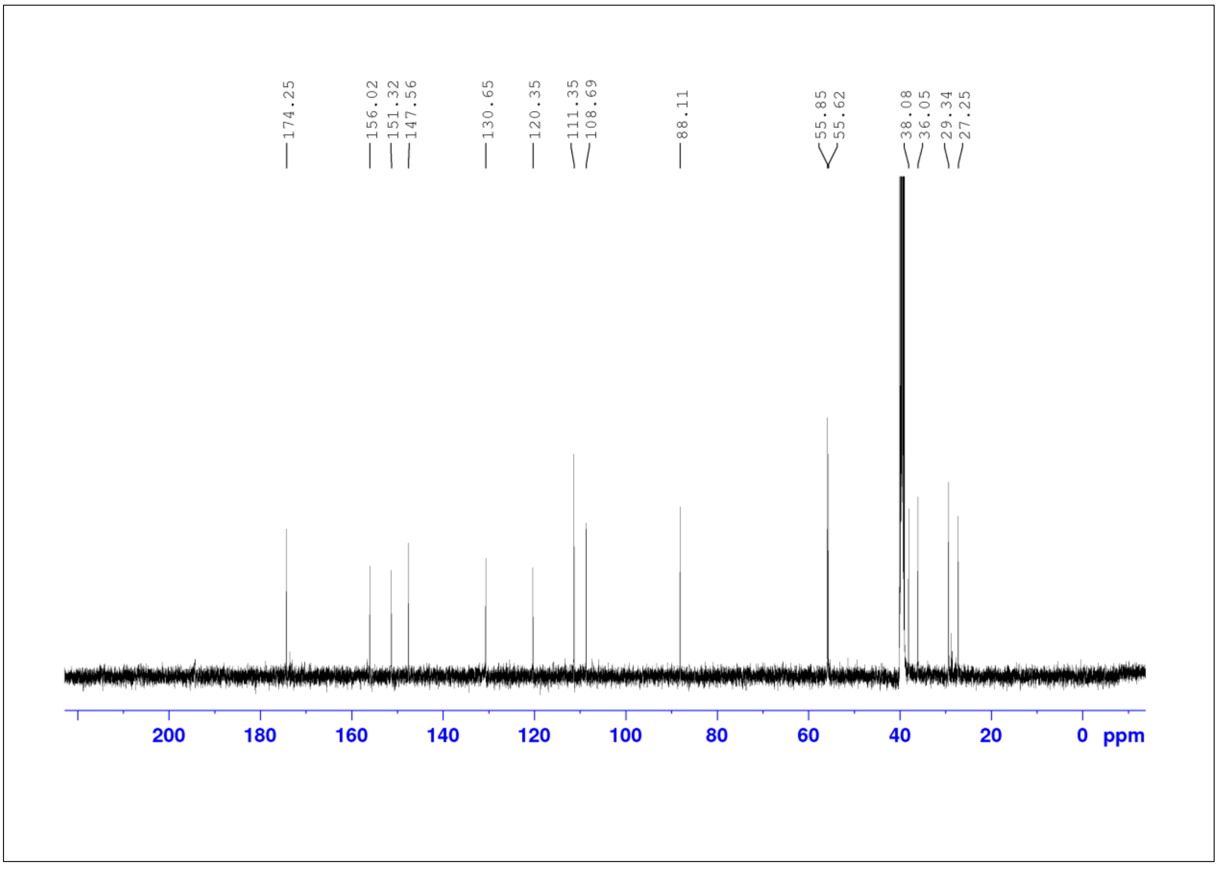

^{1}H NMR spectrum of (±)-*trans*-**28** (only one enantiomer is shown)

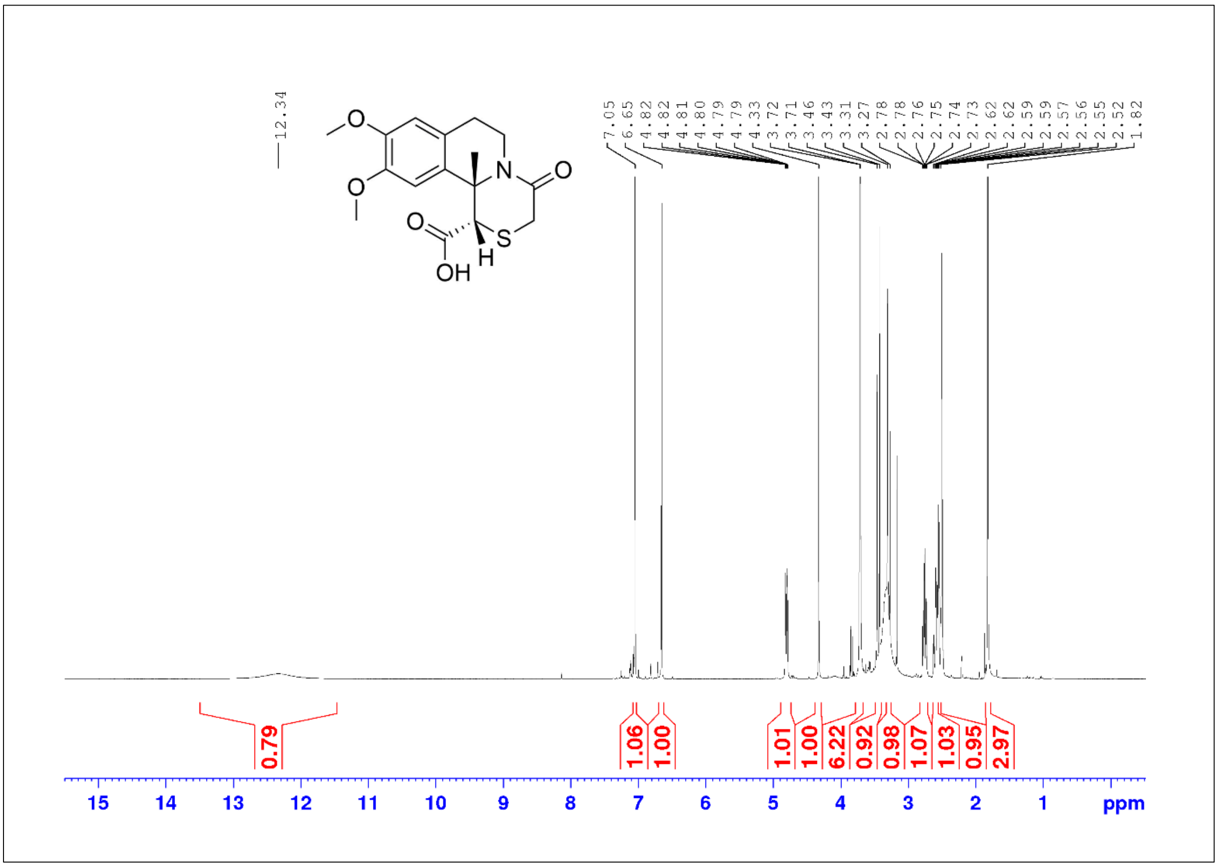

^{13}C NMR spectrum of *trans*-**28**

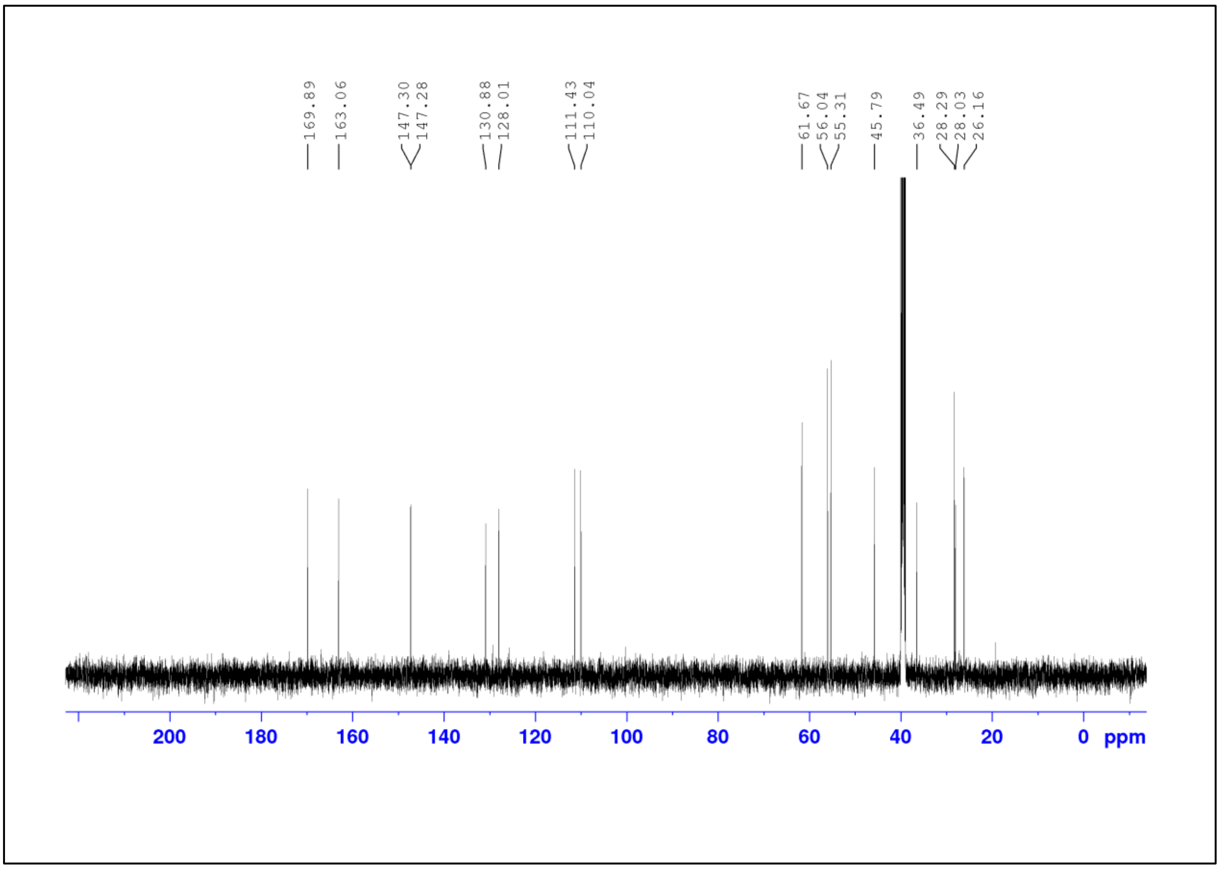

^{1}H NMR spectrum of (±)-*trans*-**29** (only one enantiomer is shown)

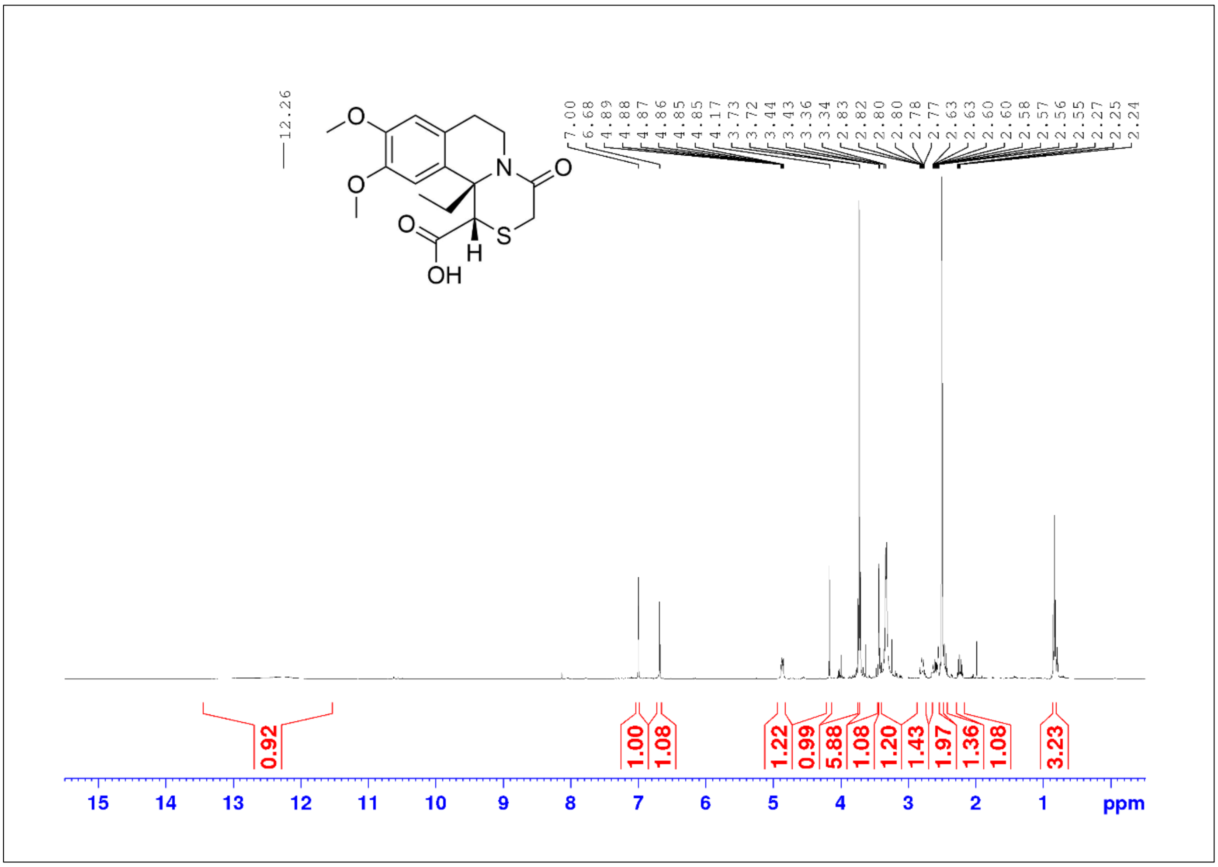

^{13}C NMR spectrum of *trans*-**29**

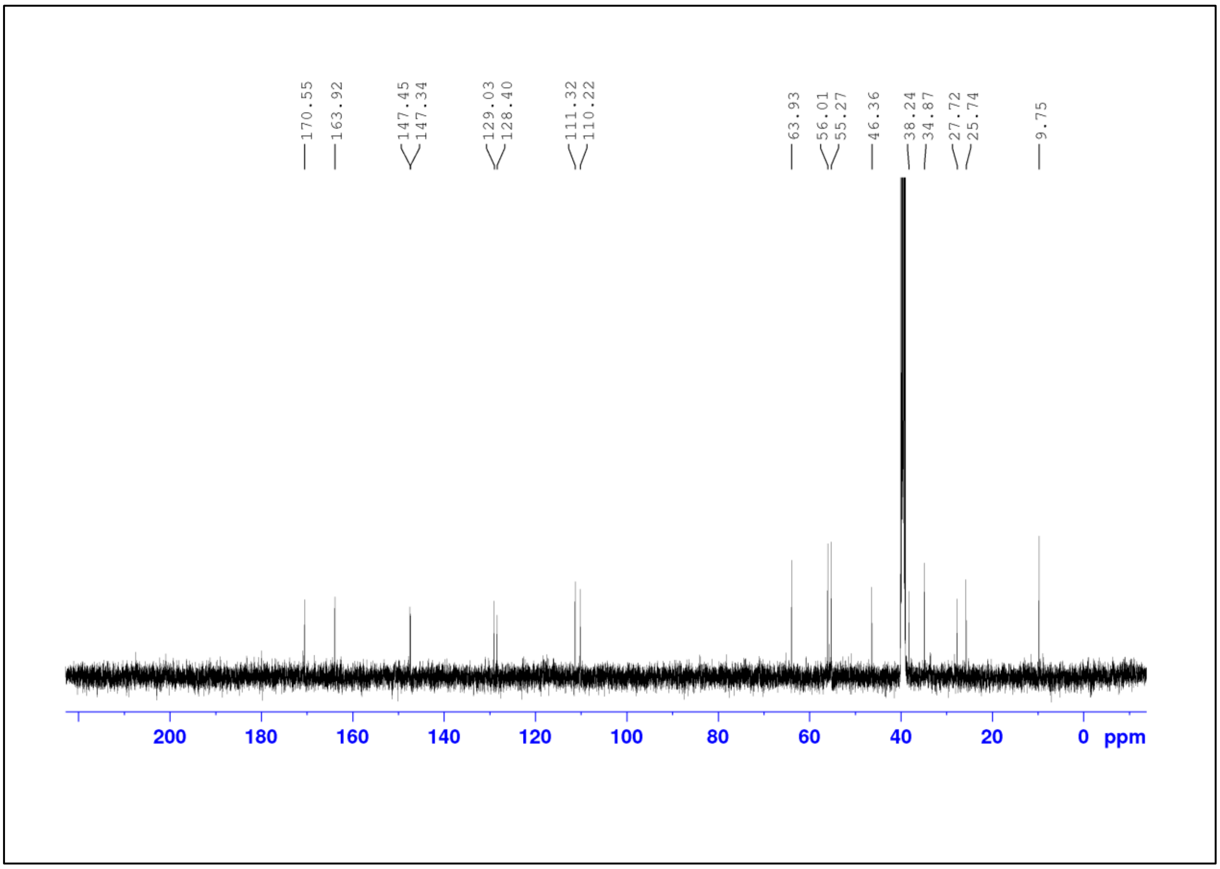

Supplement: File 1 — Experimental procedures for compounds 21–29 and their spectroscopic and analytic data. [file Beilstein_J_Org_Chem-16-1456-s001.pdf]
